# Prebiotics, Bone and Mineral Metabolism

**DOI:** 10.1007/s00223-017-0339-3

**Published:** 2017-10-27

**Authors:** Corrie M. Whisner, Luisa F. Castillo

**Affiliations:** School of Nutrition & Health Promotion, Arizona State University, 500 North 5th Street, Phoenix, AZ 85004 USA

**Keywords:** Prebiotic, Fiber, Calcium, Bone, Osteoporosis, Microbiome

## Abstract

Increasing interest in functional foods has driven discovery in the area of bioactive compounds. Prebiotics are non-digestible carbohydrate compounds that, when consumed, elicit health benefits and aid in the prevention and treatment of chronic diseases. While prebiotics have been shown to improve a number of chronic, inflammatory conditions, growing evidence exists for prebiotic effects on calcium metabolism and bone health. These novel dietary fibers have been shown to increase calcium absorption in the lower intestines of both preclinical and human models. Rodent models have also been imperative for understanding prebiotic effects on bone mineral density and measures of skeletal strength. Although fewer data are available for humans, bone-related prebiotic effects exist across the lifecycle, suggesting benefits for attainment of peak bone mass during adolescence and minimized bone resorption among postmenopausal women. These effects are thought to occur through prebiotic–microbe interactions in the large intestine. Current prebiotic mechanisms for improved mineral absorption and skeletal health include alterations in gut microbiota composition, production of short-chain fatty acids, altered intestinal pH, biomarker modification, and immune system regulation. While the majority of available data support improved mineral bioavailability, emerging evidence suggests alternate microbial roles and the presence of an intricate gut–bone signaling axis. Overall, the current scientific literature supports prebiotic consumption as a cost-effective and sustainable approach for improved skeletal health and/or fracture prevention. The goal of this review is to discuss both foundational and recent research in the area of prebiotics, mineral metabolism, and bone health.

## Introduction

Recent advances in genomic sequencing technologies have resulted in a great appreciation for the gut microbiome and its role in physiological processes that affect health. This rapidly growing area of research is demonstrating the great importance of microbes for intestinal barrier function, energy metabolism, nutrient supply, immune and inflammatory responses as well as for disease prevention and treatment [1, 2]. While many studies have linked alterations in gut microbiota structure with obesity, gastrointestinal (GI) disorders and cardiometabolic diseases [3], new methods of genomic inquiry now allow for the study of gut microbial interactions with peripheral tissues, such as bone. In fact, several mechanisms to support a gut–bone axis have recently been reviewed [4], for which the gut microbiota play an important role. This concept greatly expands our understanding of the intestine’s role in bone health beyond simply facilitating the absorption of minerals important for bone health.

**Fig. 1:**
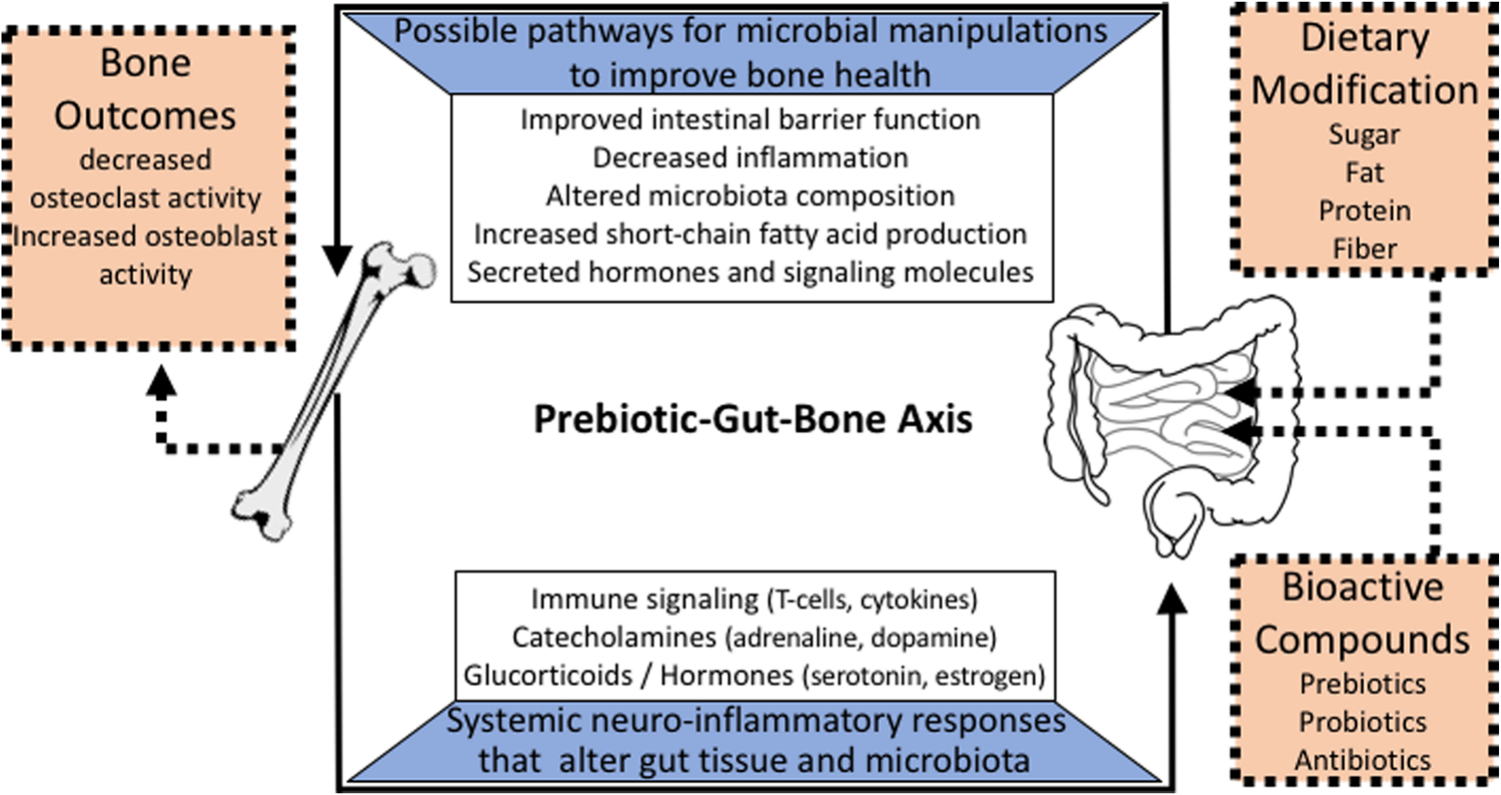
Proposed prebiotic–gut–bone axis. Dietary consumption of prebiotics stimulates gut microbial mechanisms that mediate the intestinal environment and tissue morphology. These changes promote the production of signaling molecules, immune cells, and metabolites thought to beneficially influence bone mineral uptake. Microbial signaling molecules may trigger systemic neuroinflammatory responses that ultimately stimulate the release of hematopoietic and immune stem cells from the bone marrow which feed back to the intestinal tissue to influence intestinal microbial communities and tissue inflammation

The GI tract is a highly innervated tissue which mediates digestion and absorption of dietary constituents while also facilitating communication with peripheral tissues through various microbially produced signaling molecules. Host behavior is central to these processes as the gut microbiota are dependent on dietary intake of the host for survival. Consumption of non-digestible food components is one way in which human behavior can dramatically modify the gut microbiota for improved host health. Dietary components like prebiotic dietary fiber have been linked to shifts in the gut microbial community composition. A growing body of preclinical and clinical literature shows that prebiotics are essential for improving the intestinal absorption of calcium and other minerals and also for enhancing skeletal health [5, 6]. Carefully designed clinical trials with prebiotics have resulted in a better understanding of potential mechanisms by which microbiota impact bone health, but a large gap exists to understand how prebiotics indirectly or directly manipulate the gut–bone axis (Fig. 1) to prevent or treat age-related bone diseases such as osteoporosis. Therefore, the goal of this review is to thoroughly review the current preclinical and clinical literature on prebiotic-mediated bone effects in the context of potential gut microbiota mechanisms. The clinical importance of these findings, types of prebiotics, and areas for future research will also be discussed.

## Prebiotics

**Fig. 2:**
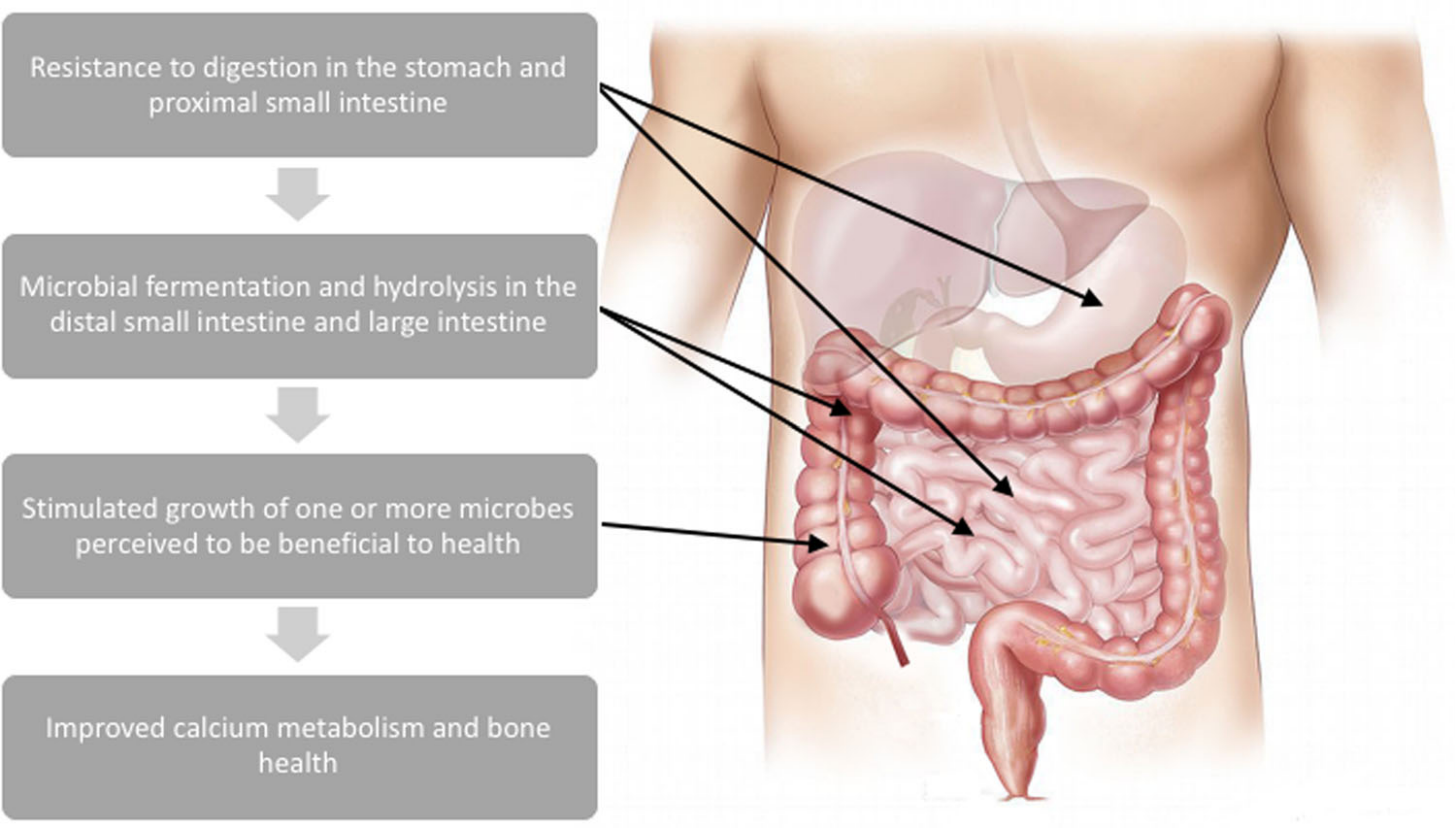
Physiological and metabolic requirements for the classification of dietary fibers with prebiotic effects

Prebiotics are functional food components which occur both naturally in plant-based foods or from synthetic production via enzymatic conversion of sugars. These compounds are generally carbohydrate structures or soluble dietary fibers which are selectively metabolized by microbes in and on the body. This action thereby supports the proliferation of specific microbes and confers a health benefit to the host (Fig. 2) [7]. With rapid growth in the area of diet–gut microbiome interactions, a new definition has recently been proposed which challenges the idea that prebiotic effects must be selective or specific [8]. This revision also suggests a need to focus on more diverse compounds (beyond carbohydrates) that influence overall microbial ecology and function which may align more clearly with host physiology [8]. Oligosaccharides like inulin-type fructans and galactooligosaccharides are the best-known fibers in this class of functional fibers and are well supported in the literature for their prebiotic effects. Specifically, these oligosaccharides have been well characterized for their ability to stimulate the growth of bifidobacteria and to a lesser degree lactobacillus [9]. Although prebiotics are classified under the umbrella of dietary fiber, the scientific concept of this more functional classification did not emerge until 1995 [10]. This definition was later modified and now expands beyond GI influences to include other microbial communities on the human body (e.g., oral, skin, urogenital) [7].

Foods containing greater amounts of prebiotics have been consumed for centuries with intakes as high as 135 g/day estimated for hunter–gatherer populations [11]. Current prebiotic consumption in Western industrialized countries is quite low in comparison with estimated daily consumption ranging from 1 to 4 g in Americans and 3–11 g in Western Europeans [12]. This is despite the presence of prebiotics in a diverse set of foods including vegetables, tubers, and grains. Prebiotic fibers are specifically prevalent in chicory root, leeks, Jerusalem artichokes, asparagus, garlic, onion, wheat, oats, and soybeans. Bananas also contain small amounts of the fructose-rich prebiotic, inulin. Although many foods naturally contain prebiotics, it is also important to consider other factors when evaluating their effectiveness, including prebiotic activity and probiotic/microbe-stimulating capacity [13].

### Prebiotics with Well-Established Bone Effects

**Fig. 3:**
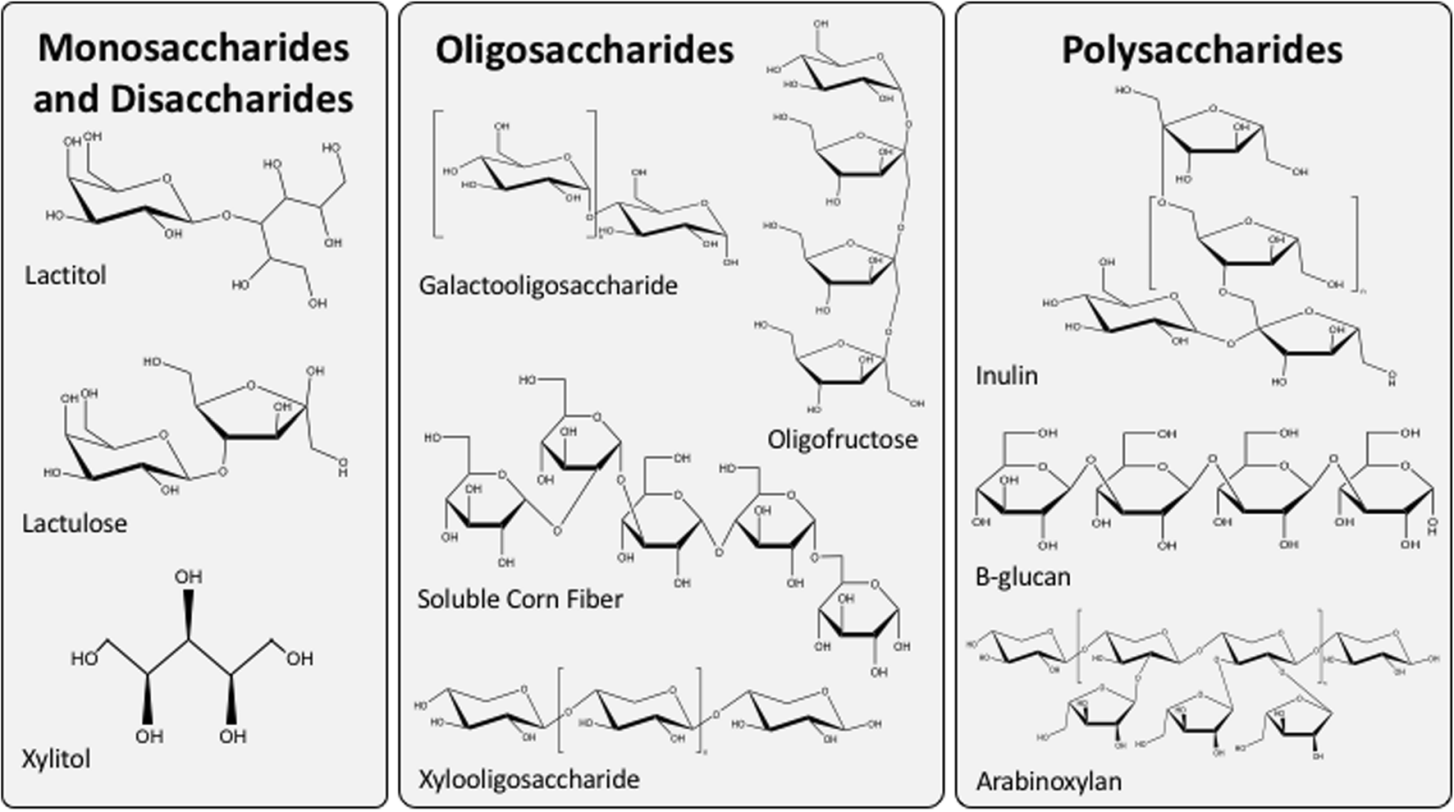
Structures of compounds with established prebiotic effects

Non-digestible oligosaccharides (NDOs) are currently regarded as the most promising prebiotics for bone health (Fig. 3). Examples of these compounds include lactulose, galactooligosaccharides (GOS), fructooligosaccharides (FOS), oligofructose, and inulin. These bioactive food components, extracts, and synthetic compounds are available with varying degrees of polymerization (DP, number of sugar monomers in each chain). Oligosaccharides make up the largest group of prebiotics with a mean DP between 4 and 10. However, disaccharides (lactitol, lactulose, etc.) and longer-chain molecules (DP 10–60) such as inulin and long-chain FOS also have prebiotic effects [14]. The area of prebiotic research has grown considerably in the past decade. Beyond the compounds mentioned above, other functional fibers and prebiotics have been identified for their positive health effects [9, 14]. With regard to bone health outcomes, the majority of available data suggest the effectiveness of FOS, inulin, GOS, lactose derivatives, and soluble corn fiber (SCF). Compounds such as xylooligosaccharides (XOS), arabinoxylans, beta-glucans, and synbiotics have been shown to have prebiotic effects but fewer data are available for their ability to influence calcium metabolism and bone health.

#### Fructans

Fructans make up the most diverse commercially available category of prebiotics which include non-digestible polymers of fructose. Their effects on calcium absorption and bone mineral density have been reviewed [14, 15]. The terminology used to describe these oligosaccharides and polysaccharides has not been well standardized but includes classifications such as oligofructose, inulin, inulin-type fructans, and FOS. A hallmark of these compounds is that the majority of glycosidic bonds in each chain are beta (β) (2–1) fructosyl-fructose linkages [16]. Fructose chains may contain a terminal glucose or consist entirely of fructose monomers. With a DP ranging between 2 and 60, fructans have been sub-classified by chain length. Short-chain fructooligosaccharides (sc-FOS) have been characterized as having a DP of 3–6 units and oligofructose a mean DP of 4. While inulin typically has an average DP of 12, long-chain FOS (lcFOS) and high-molecular weight inulin have been characterized with a mean DP of 25 [14].

These compounds can be synthetically made but also occur naturally in plant-based foods including chicory root, artichoke, wheat, onion, asparagus, agave, and banana [16]. Hot water extraction of chicory root is the most common method of producing inulin from natural sources. These extracts can be refined further into oligofructose by partial enzymatic hydrolysis via endoinulase [17] or to high-purity inulin via physical separation [17]. Commercial production of synthetic FOS results from the enzymatic conversion of sucrose, commonly by exploiting the beta-fructosidase enzyme of the fungus *Aspergillus niger* [17]. This process results in chains of fructose with β(2–1) linkages. FOS synthesis from glucose is also common but results in short-chain inulin or FOS (DP of 2–4) with a higher proportion of glucose [18].

#### Galactooligosaccharide

GOS are well-established prebiotic ingredients that result from a mixture of galactose-based oligosaccharides with varying DP and linkages to different sugar monomers including glucose and lactose. The diverse oligosaccharide composition of GOS accounts for their varied health benefits. Studies have shown that GOS fosters the growth of important gut microbes like bifidobacteria and lactobacillus during early life, as these compounds mimic human milk oligosaccharides known to promote gut health and immunity in nursing infants [19, 20].

GOS are industrially synthesized through the enzymatic hydrolysis of the glycosidic bond present in lactose by a kinetically controlled β-galactosidase reaction. The reaction is one of acid/base balance where the catalytic residues are stereochemically selected for hydrolysis [21]. The resulting galactose chain with a glucose anchor has a mean DP of 2–8 and is connected via β(1–2), β(1–3), β(1–4), and β(1–6) glycosidic linkages [22, 23]. The combination of these types of linkages between galactose units and galactose–glucose units has been associated with this prebiotic’s ability to promote the growth of specific beneficial gut microbes (e.g., bifidobacteria) [22]. Animal studies have observed that GOS consumption in postmenopausal rat models resulted in significant increases in skeletal calcium content of ovariectomized rats [24] and bone mineralization in male rats [25, 26]. Dietary GOS has also been observed to increase calcium absorption in postmenopausal women [27].

#### Lactose Derivatives

Lactose is the predominant disaccharide found in dairy products. It is composed of the sugar monomers glucose and galactose which are connected by a β(1–4) glycosidic linkage. Rats with a diet rich in lactose and calcium have been shown to have improved bone mineral content and strength [28]. As humans age, the enzymatic activity of lactase decreases, possibly allowing for microbial digestion of this sugar as it reaches the lower GI tract [29]. This may result in greater mineral absorption as lactose-intolerant individuals have been observed to experience greater calcium absorption from lactose-containing milk compared to individuals with normal lactase activity [30]. However, one study found that lactose did not increase calcium absorption among lactase-deficient individuals compared to those with normal lactase activity [31]. Lactase-deficient individuals have also been found to absorb more calcium than lactase-sufficient individuals when comparing unhydrolyzed milk to lactose-hydrolyzed milk [32]. Differences in lactase enzyme activity among individuals may be responsible for the observed differences among lactase-deficient and sufficient individuals, but the β-galactosidase activity of colonic microbiota may also play a role which ultimately challenges whether lactose should be considered a prebiotic [33].

Lactulose, a product of heat-treated lactose, has been utilized in the food, medical, and pharmaceutical industries for decades due to its beneficial interactions with gut microbes, laxation-promoting effects, and its activity as a detoxifying agent [34]. Industrial production of lactulose is performed by the isomerization of lactose in alkaline media, which results in the exchange of the glucose unit for a fructose, retaining the same β(1–4) glycosidic linkage to galactose [35]. Lactulose consumption has been shown to significantly increase calcium absorption in both rats [36] and postmenopausal women [37].

#### Soluble Corn Fiber

Soluble corn fiber (SCF) is a soluble fiber with a mean DP of 10 that is produced by the enzymatic hydrolysis of corn starch, resulting in glucose chains containing a mixture of α(1–2), α(1–3), α(1–4), and α(1–6) glycosidic linkages. SCF has a low viscosity and is resistant to processing and manufacturing techniques such as heat and variable pH [38]. The α-linkages present in SCF are indigestible in the upper GI tract, thereby allowing for microbial fermentation in the lower gut. Studies suggest that daily consumption of 8–21 g/day of SCF increases the proportions of *Bifidobacteria* in feces [39] and intakes of up to 65 g/day were better tolerated than inulin at lower doses [40]. SCF studies in animals and humans have associated this fiber with improved calcium absorption and bone strength in rats [41], greater calcium absorption in adolescent boys and girls [42, 43], and improved calcium retention in postmenopausal women [44].

#### Synbiotics

The combination of prebiotics with other bioactive ingredients such as probiotics (live microorganisms) or polyphenolic compounds is another area of greater study with regard to bone health as the combination of different products is believed to elicit a synergistic health benefit. Traditionally, the term “synbiotic” has been used to refer to the combination of prebiotics and probiotics which improve the viability of probiotics being consumed [45]. In the context of this review, we will broaden the term “synbiotic” to include more diverse combinations because other bioactive compounds also appear to elicit synergistic effects on bone health outcomes when combined with prebiotics.

One approach to synbiotics is the mixing of prebiotics of varying chain lengths and monomer linkages as this has been shown to prolong the prebiotic effect across a larger portion of the GI tract [46]. Short-chain prebiotics, such as oligofructose, are believed to be metabolized in the proximal colon, while long-chain compounds, like inulin, may be metabolized more distally or along an extended portion of the intestine. The most cited prebiotic mixture with varying chain lengths is composed of inulin-type fructans and often called ITF-mix. This mixture has been shown to have positive effects on calcium and magnesium absorption and bone health outcomes in both adolescents and postmenopausal women [47–52]. Varying the type of prebiotics to include different sugar monomers has also been shown to be effective as greater trabecular bone mineral density, bone volume, osteoblast surface area, and measures of stiffness and elasticity have been observed in growing rat models following the consumption of a GOS and FOS mixture [53].

Synergistic effects of prebiotics with plant polyphenols have broad applicability for bone health as well. One study reported improvements in trabecular microarchitectural properties of the tibia despite no change in BMD following treatment with a FOS and soy isoflavone mixture in ovariectomized rats [54]. FOS combined with dried plum fractions in soy-based diets have also resulted in greater whole-body BMD in ovariectomized rats [55].

Synbiotic investigation that evaluates the effectiveness of prebiotics in combination with probiotics is a rapidly growing research area. Currently, *Bifidobacterium* species are the best studied in synbiotic applications with regard to bone health. FOS combined with the bacterial species, *Bifidobacterium longum*, increased the calcium, magnesium, and phosphorus content of bone as well as bone-breaking force in rats [56]. Another study reported that combining two species, *Bifidobacterium bifidum* and *longum*, with GOS significantly increased calcium, magnesium, and phosphorus bioavailability and hind limb bone mineral content [57, 58].

Dairy products, especially fermented milk products, have also been investigated for their synergistic effects on mineral and bone metabolism. Fermented milk consumption before sleeping for 2 weeks among postmenopausal women resulted in decreased bone resorption [59]. Despite the addition of inulin-type fructans and caseinophosphopeptides to the fermented product, no additional effect was seen on bone resorption; however, this supplemented group resulted in greater urinary excretion of calcium and phosphorus which may be the result of increased intestinal absorption [59]. Another study of kefir-style fermented milk had no effect on bone turnover, as measured by biochemical blood and urine markers, but this may have been due to the intervention product being supplemented with 1600 mg of calcium carbonate [60]. FOS–inulin-supplemented milk consumption for 12 weeks among both pre- and postmenopausal women resulted in significant reductions in bone resorption (decreased urinary excretion of C-telopeptide of type I collagen) among postmenopausal but not premenopausal women [61].

### Potential Candidates for Prebiotic–Bone Interactions

A number of other dietary fibers and sugar alcohols have been evaluated for their prebiotic effects in the gut and subsequent health benefits in relation to metabolic conditions including diabetes, cardiovascular disease, obesity, and metabolic syndrome. While these prebiotics have not been extensively investigated for their effects on mineral metabolism and bone outcomes, it is possible that they also elicit similar effects on mineral bioavailability and bone health parameters. Therefore, a future area of investigation would be to evaluate the effectiveness of these compounds and compare their effects to prebiotics with already-established bone effects.

#### Beta-Glucans

Beta-glucans (β-glucans) are polysaccharides of d-glucose monomers linked by β-glycosidic linkages. This well-studied dietary fiber is found in cereal grains, yeast, mushrooms, seaweeds, and some bacteria. Chemically, β-glucans are non-starch polysaccharides with repeating glucose units that may or may not contain branch points. The prebiotic benefits of β-glucans have been reviewed and primarily highlight their blood lipid-lowering effects [62]. Among finisher pigs, β-glucans have been shown to increase short-chain fatty acid production in the intestine and promote the growth of beneficial bacteria such as bifidobacteria, but no effect of this prebiotic was observed for calcium and phosphorus digestibility or retention [63]. Further work is needed to evaluate the diverse types of β-glucans currently available in the food supply and to see if these effects on calcium metabolism also occur in other animal models, as well as in humans.

#### Arabinoxylans

Arabinoxylans (AX) are an important contributor to fiber consumption in the Western diet, as they are the main non-starch polysaccharides found in many cereal grains. They consist of β(1,4) linked d-xylopyranosyl residues to which arabinofuranosyl moieties are attached. Partial enzymatic hydrolysis of AX yields arabinoxylan oligosaccharides (AXOS) and xylooligosaccharides (XOS) which have been evaluated for their prebiotic and health effects in a recent review [64]. Specifically, AX has been associated with greater fecal bifidobacteria content [65].

#### Xylitol

Xylitol is an emerging prebiotic that has shown promising applications in food, pharmaceutical, and medical industries as a low-calorie sweetener. Xylitol is a non-digestible pentose sugar with an alcohol moiety. Manufacturing of this prebiotic results from the extraction from lignocellulosic materials (polymers of cellulose, hemicellulose, and lignan) such as birchwood [66]. In whole foods, xylitol is found naturally in many fruits and vegetables [66]. Xylitol has been observed to promote the proliferation of lactobacilli and bifidobacteria while decreasing the presence of pathogens in the intestines [66].

#### Xylooligosaccharides

Xylooligosaccharides (XOS) are non-digestible oligosaccharides with a mean DP of 2–6 and β(1–4) glycosidic bonds that also result from the direct enzymatic auto-hydrolysis of xylan-rich lignocellulosic materials [67]. XOS are indigestible in the upper part of the GI tract, thereby affecting the host by selectively stimulating the growth or activity of bacteria in the colon. XOS have been shown to increase cecal bifidobacterial counts and total anaerobic bacteria in the lower gut of rats fed non-digestible oligosaccharides which included a XOS treatment [68]. In comparison to other prebiotic oligosaccharides, XOS resulted in greater colonic total wet weight but had no effect on colon wall weight [68]. Interestingly, a very recent investigation of dietary XOS on bone mineral crystallinity in swine femurs suggests that XOS increases the crystallinity of bone and that the influence of XOS on bone may be more prevalent during periods of growth, as the effects were not seen in bones collected during later stages of life [69].

## Local Intestinal and Systemic Effects of Prebiotics

Diet is a well-accepted contributor to bone health but how prebiotics interact with the microbiome to impact bone remains poorly understood. Intestinal microbiota represent an immensely rich community of bacteria, fungi, viruses, and archaea that collectively contribute more than 3 million unique genes [70], the activities of which are far-reaching as evidenced by the excretion of more than 200 microbially produced metabolites in urine [71]. While it remains difficult to separate host gene actions from those of the gut microbiota following prebiotic consumption, it is clear that the gut microbiome has large impacts on host metabolic actions through stimulated immune responses, increased digestion and absorption of dietary components, displacement-mediated pathogen inhibition, and/or improved intestinal barrier function. The following section provides a summary of data to support current mechanistic theories mediated by prebiotic–microbiome interactions.

### Microbial Community Composition

The interaction between gut microbiota and skeletal tissue became apparent when ovariectomized rats were fed antibiotics, or antibiotics in the presence of prebiotics or polyamines [72, 73] and very recently when germ-free mice treated with feces from conventionally raised mice experienced a decrease in trabecular bone mineral density, trabecular bone volume, trabecular number, and cortical bone area [74]. Inoculation with microbiota also increased the total number of osteoclasts relative to control germ-free mice. While these findings suggest a microbial role in bone catabolism, prebiotics appear to elicit an anabolic effect via the microbiome. To date, very few studies have evaluated a link between prebiotic-induced microbial changes in the gut with improvements in calcium absorption and/or markers of bone health. Of the animal studies that have explored this interaction, bifidobacteria and *Bacteroides* have been identified as mediators of calcium absorption, intestinal morphological and pH changes, and improved bone strength [26, 75–77]. Two recent studies of adolescent calcium absorption which aimed to correlate improvements in calcium absorption with changes in the gut microbiota found that SCF increased *Bacteroides*, *Butyricicoccus*, *Oscillibacter*, *Dialister, Parabacteroides, and Clostridium* [42, 43]. With age, the gut microbiome changes, so whether the same microbial genera associated with calcium absorption during adolescence persist to elicit the same effects later in life (e.g., postmenopausal period) remains an open question. Largely, the bacterial groups that have currently been identified for their role in calcium absorption are known for their starch and fiber fermentation capabilities which supports the following mechanisms.

### Short-Chain Fatty Acids (SCFAs) and Luminal pH

**Fig. 4:**
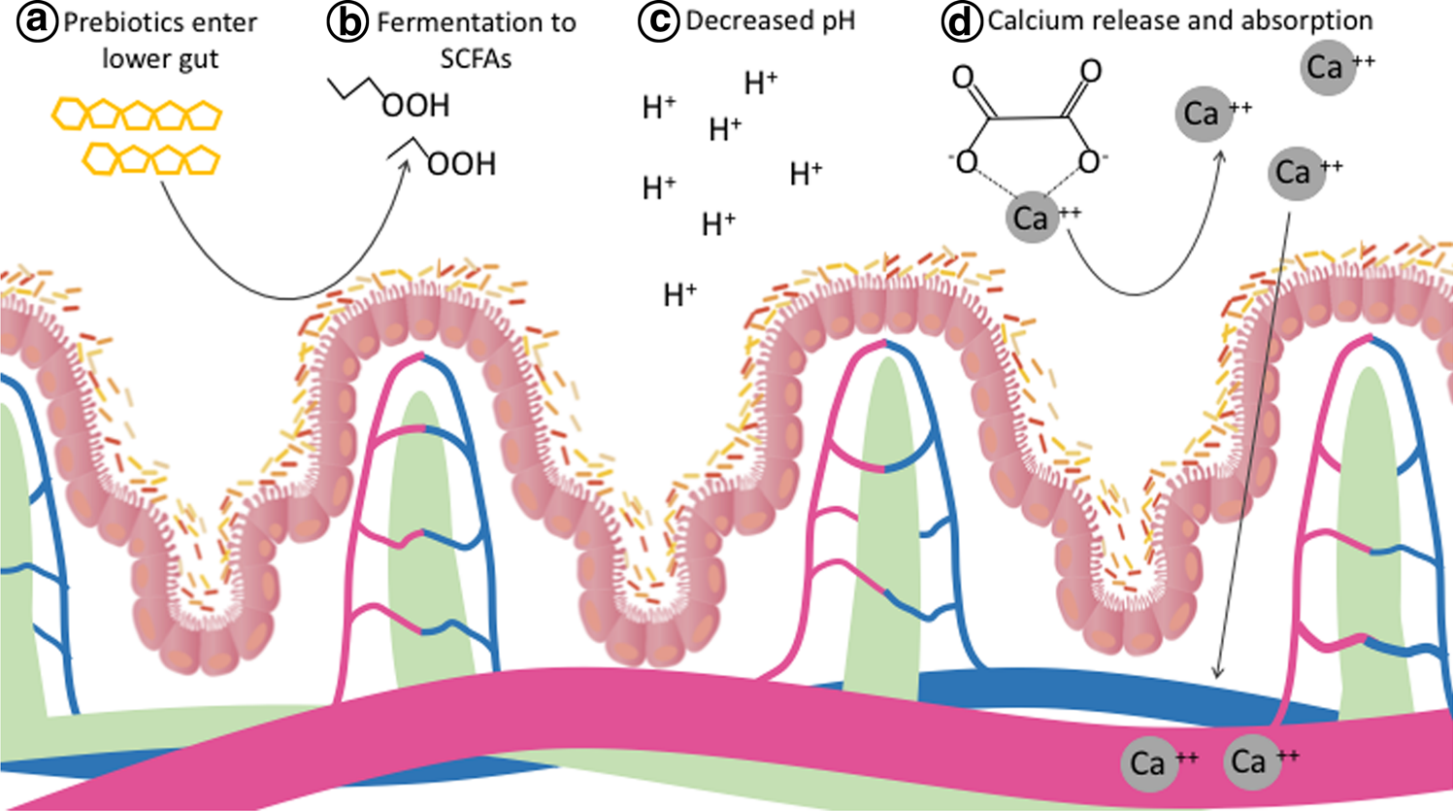
A primary mechanism to explain prebiotic benefits on calcium absorption is through (*a*) fermentation of prebiotics by saccharolytic microbes. This results in (*b*) the production of short-chain fatty acids and (*c*) a reduction in intestinal pH. These actions are believed to (*d*) ionize calcium from negatively charged compounds in the colon, thereby allowing calcium to absorb into the blood. *H+* protons, *SCFAs* short-chain fatty acids

Currently, the most widely accepted theory explaining prebiotic–bone interactions is through the production of short-chain fatty acids (SCFAs) in the lower gut (Fig. 4). Microbial fermentation and hydrolysis reactions transform prebiotics to SCFAs, thereby lowering the pH of intestinal luminal contents [73]. Greater acidity in the colon is thought to prevent calcium from complexing with negatively charged metabolites including phytates and oxalates. The release of calcium from these molecules increases the mineral’s availability for absorption and subsequent bone mineralization. Prebiotic fibers have been shown to increase the cecal content of SCFAs (acetate, propionate, butyrate, isobutyrate, valerate, and isovalerate) in animal models [41, 78, 79]. Despite observed changes in intestinal SCFA production, a few animal studies designed to observe prebiotic-associated increases in these metabolites reported different findings: While one study found no change in SCFA production [80], another study that observed increases found only weak associations between cecal SCFA concentrations, calcium absorption, and bone mineral density [41]. Intestinal chamber experiments comparing calcium absorption following exposure to SCFA or hydrochloric acid, an attempt has been made to identify whether SCFAs or pH is responsible for the observed bone effects. Findings suggested that only the SCFA treatment increased calcium transport across colonic cells [81]. This finding provides support for alternative roles of SCFAs in cellular proliferation and cell signaling pathways as discussed below.

### Changes in Tissue Morphology and Transport Proteins

**Fig. 5:**
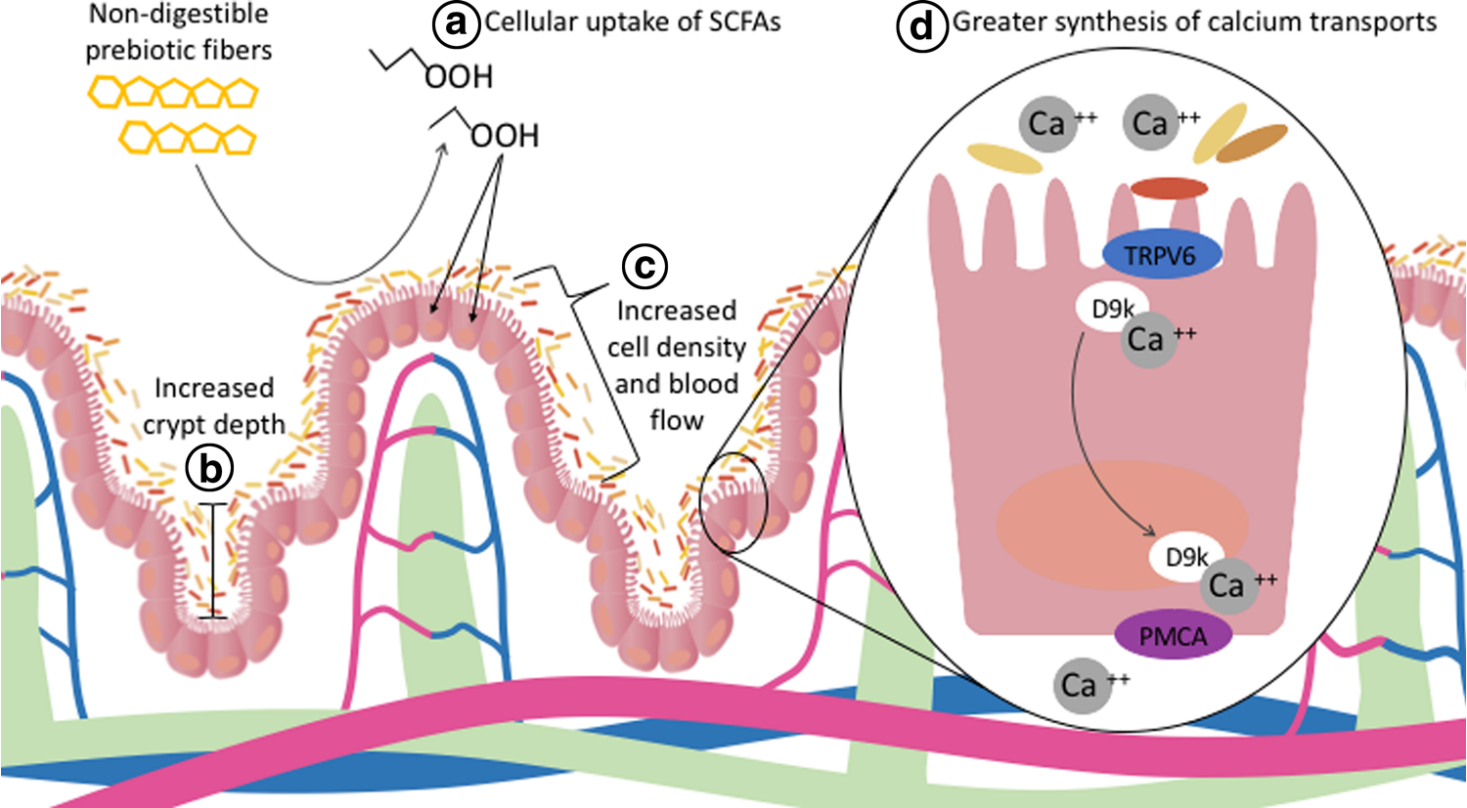
Prebiotics influence intestinal morphology and the presence of calcium transport proteins. This mechanism involves (*a*) fermentation of prebiotic fibers by saccharolytic microbes in the large intestine to form SCFAs such as butyrate and acetate. Cellular uptake of SCFAs increases cell proliferation ultimately resulting in (*b*) increased intestinal crypt depth and (*c*) greater cell density and blood flow in the villi. This increase in mucosal tissue provides greater surface area for calcium absorption. Additionally, microbial metabolites such as SCFA may signal for (*d*) greater gene expression of the intracellular calcium transporter calbindin D9k. *D9k* calbindin D9k, *PMCA* plasma membrane Ca^2+^-ATPase, *SCFAs* short-chain fatty acids, *TRPV6* transient receptor potential cation channel subfamily V member 6 calcium-specific transport protein

Increases in cell density, intestinal crypt depth, and blood flow have been observed in the lower gut following prebiotic consumption (Fig. 5), which are believed to increase intestinal surface area and allow for greater mineral absorption [73, 79]. A possible reason for these morphological changes is greater SCFA production, as butyrate is the preferred energy source for colonocytes [82]. Gut microbes synthesize a large portion of intestinal SCFAs which may explain the link between gut microbiota and increases in intestinal mucosal cell proliferation [83]. With regard to bone health, morphological changes following prebiotic consumption have been associated with increases in calcium absorption in animal models [79]. Beyond physical changes observed at the tissue level, changes at the cellular level have also been observed. In animal models, prebiotic consumption has been shown to increase cecal and colon expression of Calbindin D9k, an intracellular calcium transport protein [84]. Regulation of mineral transport proteins may be the result of gene expression, as SCFAs are believed to be involved in epigenetic modifications of the DNA structure [85].

### Hormonal and Immune Signaling

**Fig. 6:**
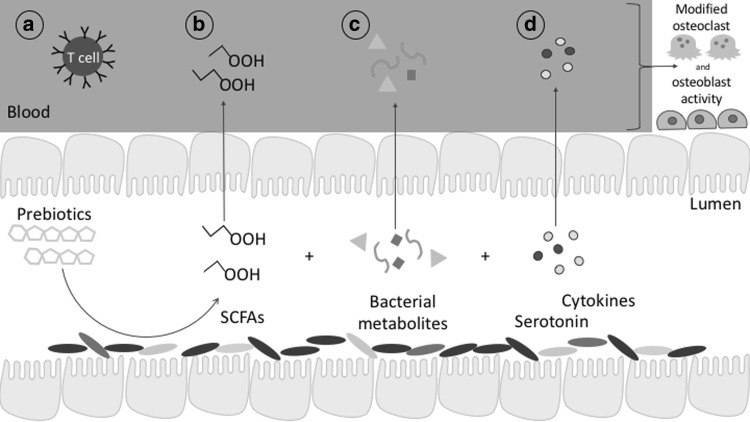
Emerging gut microbial mechanisms that may also benefit bone. Microbial interactions with prebiotics may modify (*a*) immune cell synthesis; (*b*) short-chain fatty acid uptake; (*c*) production of beneficial or inhibitory microbial metabolites; and (*d*) production of serotonin and cytokines. Presence of these metabolites in the systemic circulation may influence gene expression in bone cells and also interact with neural signaling pathways which further affect bone tissue via immune signaling. *SCFAs* short-chain fatty acids

Alternative hormonal and immune signaling mechanisms (Fig. 6) may explain further the link between SCFAs and bone health, but at this time data are not available to directly link these pathways with prebiotic-induced bone outcomes. Gut microbiota are believed to interact with the immune system as evidenced by germ-free mice presenting with reduced mucosal immune function and signaling [74, 86]. Beyond the gut, systemic inflammation and disrupted immune signaling have been linked to decreased bone mass [87]. This link with bone may arise from activated T cells stimulating the production of TNFα in bone marrow and subsequent osteoclastogenesis [5].

SCFAs may also be involved in hormonal signaling that affects bone, as specific gut microbial strains have been linked to peripheral serotonin production [88]. In vitro studies of osteoblast cells suggest that serotonin signaling activates the serotonin 6 G-protein-coupled receptor (5-HT_6_R) which inhibits bone mineralization possibly by downregulating alkaline phosphatase [89]. When gut microbial production of serotonin was inhibited, ovariectomized rats developed significantly more bone mass [90]. Currently, no studies have evaluated the effects of prebiotics on gut microbe-derived serotonin and whether such hormones interact with skeletal tissue, but emerging data clearly link gut microbial activities with bone health outcomes. Prebiotics do stimulate the proliferation of beneficial gut microbes which could be a target for promoting the production of beneficial metabolites for bone health, including immune and hormonal signaling molecules.

## Evidence from Preclinical Models

The majority of literature to support the effects of prebiotics on bone comes from animal research with a small portion from in vitro cell studies. Together, this literature provides the strongest support for mechanisms of action while also characterizing interactions between prebiotic type and calcium dose when evaluating bone outcomes [91, 92]. Preclinical studies also provide ample evidence for prebiotic effects on bone throughout the life course as evidence is available from young, adult, and elderly animal models. Despite the depth and breadth of the animal literature, few studies have integrated gut microbial community data with bone health outcomes in an attempt to model/elucidate how prebiotics influence gut–bone interactions [6]. A summary of animal studies can be found in Table 1.

**Table 1:** A summary of prebiotic interventions among animals to evaluate their effectiveness in altering mineral absorption, bone health outcomes, and intestinal parameters

Prebiotic/substance	Treatment dose and duration	Study design	Animal model description	Measures/analyses	Findings	Abbreviated reference
*Anoectochilus formosanus* (AF), inulin	4 diets: control, 200 mg/kg AF, 400 mg/kg AF and 400 mg/kg inulin, for 12 weeks	Parallel design with 5 groups: OVX or SHAM on control diet + water; remaining 3 OVX groups randomly assigned to supplemented diets; AF delivered as aqueous solution	Female Wistar rats (*n* = 40), age 3 months	Apparent Ca absorption and retention; tibia BMD/BMC; bone-breaking strength of femur; trabecular bone microarchitecture; fecal microbial culture assessments; cecum morphology, SCFA and pH	400 g/kg AF and 400 g/kg inulin resulted in greater apparent Ca absorption and retention, cecal wall weight and lower cecal pH, greater total fecal SCFA and butyrate, as well as improved biomechanical properties of bone and decreased bone turnover; AF improved fecal bifidobacteria content	Yang L, et al. 2013 (Ref. 78)
Difructose anhydride III (DFAIII)	0 or 15 g/kg DFAIII, for 4 weeks	Parallel design; rats divided into 4 groups (2 groups of OVX and 2 groups of SHAM rats) and randomly assigned to control (AIN93G) or vitamin D-deficient diets for 8 weeks; at 11-weeks of age, the 4 groups were further divided into DFAIII groups	Female OVX Sprague Dawley rats (*n* = 64), age 3 weeks	Apparent Ca absorption and retention; femur Ca, Mg and P content; intestinal Ca transporter expression; cecum morphology, pH and SCFA content	Serum Ca concentration and calbindin D9k mRNA levels were affected by vit-D-deficient diets; Ca absorption rates in vit-D-deficient groups were much lower than any other group; DFAIII increased Ca absorption rates in both SHAM and OVX groups, but the effect was higher in the OVX rats; femoral Ca content was significantly lower in the vit-D-deficient group in both SHAM and OVX but DFAIII increased femoral Ca content over other groups; femur Mg content was higher in the DFAIII-vit-D-deficient SHAM rats; femoral P content was influence by vit-D deficiency and OVX but not DAFIII; cecal weight was affected by vit-D deficiency, OVX and DFAIII diet; cecal pH decreased while total SCFA increased with DFAIII diets	Mitamura R, et al. 2006 (Ref. 106)
FOS	0 or 5% FOS, for 23 days	Parallel design; AIN-93G used as base diet with 7.5 g/kg Ca; FOS replaced sucrose in the diet	Male Wistar rats (*n* = 16), age 4 weeks	Apparent Ca and Mg absorption for 5 day intervals starting at days 4, 10 and 16; femur and tibia BMD by DXA; bone biomechanical properties; cecal weight and pH	Ca and Mg absorption and retention were greater with FOS compared to control at all three measurement time points even though the percentage of Ca and Mg absorbed decreased over time; femur and tibia Ca content were greater in the FOS group; no difference in BMD between the two groups; peak breaking force of femur improved with FOS; cecal wall and content weights were greater in FOS group and cecal pH was lower with FOS	Lobo A, et al. 2006 (Ref. 113)
FOS	0 or 5% FOS, for 15 days	Parallel design, SI; rats were pair-fed to match dietary intake; FOS consisted of 34% 1-ketose, 53% nystose and 10% 1F-b-fructofuranosyl nystose; diet run-in for 6 days; FOS replaced dietary sucrose	Male Wistar rats (*n* = 16), age 45 days	Apparent and true Ca absorption and kinetics	FOS increased both true Ca absorption and Ca balance relative to controls; no differences in bone formation or resorption were observed; urinary Ca excretion in FOS group was significantly greater than in controls; Ca balance in FOS group was correlated with Ca absorption	Morohashi T, et al. 1998 (Ref. 109)
FOS	0 or 50 g FOS/kg of diet, for 28 days	Parallel design; sham and cecectomized rats received both diets	Male Sprague Dawley rats (*n* = 28), age 5 weeks	Apparent Ca and Mg absorption; cecal pH and fecal SCFA concentrations	FOS improved both Ca and Mg absorption in the sham animals but only Mg in the cecectomized rats; FOS decreased cecal and colonic pH and altered the composition of SCFA	Ohta A, et al. 1994 (Ref. 107)
FOS	0 or 50 g FOS/kg of diet, for 8 days	Parallel design; 1 week dietary run-in on control diet	Male Sprague Dawley rats (*n* = 28) 6-week-old	Apparent Ca and Mg absorption	Ca and Mg absorption increased across the colon and rectum	Ohta A, et al. 1995 (Ref. 110)
FOS	100 g/kg FOS or sucrose by weight of diet, for 10 days	Parallel design; 3 study groups: sham-operated rats on control diet and gastrectomized rats distributed into each treatment group	Male Sprague Dawley rats (*n* = 17), age 4 weeks	Apparent Ca, P and Mg absorption; Ca transporter quantification by western blots	FOS-supplemented gastrectomized rats experienced improvements in apparent Mg absorption relative to gastrectomized and sham controls; FOS + gastrectomy resulted in greater P absorption relative to sham but not gastrectomized controls; Ca absorption was improved in FOS + gastrectomy rats compared to gastrectomized but not sham controls; FOS + gastrectomized rats had greater cecal mucosa wet weight and presence of calbindin D9k in the distal small intestine, cecum and colon	Ohta A, et al. 1998 (Ref. 84)
FOS	0, 50 or 100 g/kg FOS, for 10 days	Parallel design; control diet contained 100 g/kg sucrose	Male Sprague Dawley rats (*n* = 18), age 5 weeks	Apparent Ca absorption; total and calbindin D9k protein expression by Western blot	Ca absorption increased dose-dependently with FOS; small intestinal and cecal weight increased with FOS; calbindin D9k concentration was significantly increased in the cecum on the 10% FOS treatment compared to 5% FOS	Ohta A, et al. 1998a (Ref. 101)
FOS	5 and 10% nystose, 5 and 10% kestose, 5 and 10% FOS, 24 days	Parallel design; 7 study diets with control diet containing 100 g/kg sucrose, all prebiotic diets contained 50 g/kg sucrose	Male Sprague Dawley rats (*n* = 49), age 5 weeks	Apparent Ca and Mg absorption at 10 and 24 days	Each prebiotic treatment increased Ca and Mg absorption dose-dependently after 10 days but not at 24 days; 10% FOS decreased fecal Ca excretion and increased urinary Ca excretion; apparent absorption of Ca in rats on 10% FOS was higher that control rats; fecal Mg excretion decreased with 10% FOS while urinary Mg excretion increased with 10% FOS; apparent absorption of Mg was higher than controls for both 5 and 10% FOS	Ohta A, et al. 1998b (Ref. 103)
FOS	0 or 5 g FOS/100 g diet, for 15 days	Parallel design; control diet was AIN-93; FOS replaced dietary sucrose; run-in diet for 3 days before intervention; Ca content of both diets was ~ 5.2 g/kg of diet	Male Wistar rats (*n* = 16), age 45 days	Apparent and fractional Ca and Mg absorption; femur microarchitecture	Apparent and fractional Ca and Mg absorption were increased with FOS relative to controls; trabecular bone volume in the distal metaphysis and bone volume in the femoral neck were greater with FOS; bone area of the mid-femur did not differ; significant positive correlation between absorbed Ca and femoral Ca content	Takahara S, et al. 2000 (Ref. 112)
FOS and soy isoflavones (IF)	0 or 7.5% FOS with 0, 10, 20, 40, or 80 µg/g body weight of IF, for 90 days	Parallel design; 12 study groups including sham and OVX controls with and without FOS; short-chain FOS given at 2.5% for week 1, 5% for week 2 and at 7.5% for remainder of 3 months	Female OVX Wistar rats (*n* = 96), age 3 months	BMD and body composition by DXA; femur biomechanical properties; bone biomarkers	FOS enhanced the bone-sparing effects of IF by increasing femur BMD and bone strength (femur failure load); FOS addition did not significantly alter osteocalcin and deoxypyridinoline concentrations	Mathey J, et al. 2004 (Ref. 115)
FOS and soy isoflavones (IF)	5% FOS, 0.2% IF, 5% FOS + 0.2% IF, for 6 weeks	Parallel design; 2 week adaptation period before surgery; diet treatments began 7 days post-OVX; control diet was AIN-93G with corn oil replacing soybean oil	Female OVX ddY mice (*n* = 64), age 8 weeks	BMD and BMC by DXA; femur BMC by pQCT; femur Ca, Mg, and P content	Relative to OVX controls, IF and FOS + IF increased BMD of the middle and distal femur while FOS + IF increased BMD of the proximal femur; FOS + IF increased trabecular BMD of the femur compared to control and FOS alone; IF improved femur mineral content	Ohta A, et al. 2002 (Ref. 117)
FOS from yacon flour + *Bifidobacterium longum*	15.6 g yacon flour/100 g of diet, for 28 days	Parallel design; control diet consisted on AIN-93G; 4 diets: control, yacon flour, control + B. longum, yacon flour + B. longum; yacon flour contains 4% FOS	Male Wistar rats (*n* = 32)	Bone biomechanical properties; mineral content (Ca, P, Mg); cecal morphology, pH and SCFA content; bifidobacteria, lactobacillus and total anaerobes quantified	Both control + *B. longum* and yacon + *B. longum* resulted in greater tibial Ca, Mg and P content relative to control; Ca and Mg were also higher in the yacon + *B. longum* groups compared to yacon only; peak breaking force did not differ; cecal weight was greatest with the yacon diet and was also greater in yacon + *B. longum* compared to control; cecal propionate content was greater in all treatments compared to control; cecal weight and total anaerobes were highest with yacon diet	Rodrigues F, et al. 2012 (Ref. 56)
FOS, dried plum, soy	5% FOS, for 60 days	Parallel design with 6 groups: sham-operated and OVX (OVX) groups served as controls consuming casein, remaining 4 OVX groups consumed all other non-casein diets; 45 day run-in on standard diet following OVX; 5 diets: casein-based diet (casein), soy-based diet (soy), soy-based diet with dried plum at 7.5% (soy + plum), FOS at 5% (soy + FOS), and combination of dried plum and FOS (soy + plum + FOS)	Female OVX Sprague Dawley rats (*n* = 72), age 3 months	Bone biomarkers; femoral strength; DXA; histomorphometry of tibia	Higher whole-body BMD was observed in rats from soy + FOS, soy + plum, and soy + plum + FOS groups; soy + FOS increased peak breaking force of the femur more than any other treatment; reductions in bone resorption (deoxypyridinoline) and increases in alkaline phosphatase were greatest for soy diets with plum, FOS and plum + FOS relative to controls	Johnson C, et al. 2011 (Ref. 55)
FOS, soy	5% FOS, for 4 months	Parallel design with 5 groups: sham-operated and OVX groups served as controls, remaining 3 OVX groups consumed soy-based diet (soy), FOS-supplemented diet (FOS), or soy-based plus FOS (soy + FOS); 3 month run-in on standard diet following OVX	Female Sprague Dawley rats (*n* = 63), age 9 months	BMD and BMC by DXA; bone biomechanical and microarchitectural analysis	Whole-body BMD was higher in FOS and soy + FOS groups; FOS and soy + FOS diets improved lumbar BMC and BMD; soy + FOS was most effective at increasing trabecular number and decreasing trabecular separation when compared to OVX controls; femur biomechanical measures were not changed by any diet	Devareddy L, et al. 2006 (Ref. 54)
GOS	0 or 5% GOS, for 30 days	Parallel design; both diets fed to sham and OVX animals; AIN-76 diet used with GOS replacing sucrose	Female OVX Wistar rats (*n* = 36), age 4 weeks	Apparent Ca absorption; femur and tibia Ca content; cecal pH, wall and content weights; fecal SCFA concentrations	GOS improved Ca absorption at 8–10 days and 18–20 days relative to control OVX animals but this effect disappeared by 28–30 days; among the OVX group, GOS only improved tibia Ca content; cecal content and wall weight were increased by GOS and cecal pH was reduced; total SCFA and acetate, propionate, butyrate and succinate were increased with GOS treatment	Chonan O, et al. 1995 (Ref. 24)
GOS	5 g GOS/100 g of diet, for 30 days	Parallel design, SI; rats were acclimated to their assigned calcium intake for 30 days prior to GOS feeding; diets based on AIN-76 formulation; 4 diets: normal-Ca (0.5 g/100 g), low-Ca (0.05 g/100 g), and normal and low Ca with the addition of 6′-GOS	Male Wistar rats (*n* = 32), age 4 weeks	Apparent Ca absorption; femoral and tibia Ca content; cecal morphology and SCFA content	GOS was more potent than control diet at stimulating Ca absorption with normal-Ca diet after 8–10 and 18–20 days, but this effect did not persist through 28–30 days; femur and tibia Ca content were higher in rats fed GOS + normal-Ca diet; Ca absorption and bone Ca content were not affected by GOS when combined with a low-Ca diet; GOS feeding increased, cecal tissue and content weights, total SCFA and acetic, propionic and butyric acid content on both low and normal-Ca diets	Chonan O, et al. 1996 (Ref. 25)
GOS	0, 2, 4, 6 or 8% of GOS by weight of diet, for 8 weeks.	Parallel design, SI; dietary adaptation with AIN 93-G for 12 days; GOS replaced cornstarch in diet	Male Sprague Dawley rats (*n* = 75), age 4 weeks	Apparent absorption/retention of Ca and Mg; femur Ca uptake and micro-CT; cecal morphology, pH, wall and content weights; PCR-DGGE and bifidobacteria qPCR	Dose-dependent increases in net Ca and Mg absorption and retention, greater femur Ca uptake, vBMD and breaking force; dose-dependent increase in cecal wall and content weights and decreased cecal pH; PCR-DGGE profiles suggested differences in bacterial community structure and greater bifidobacteria by qPCR	Weaver C, et al. 2011 (Ref. 26)
GOS, *Bifidobacteria bifidum and longum*	0, 12, 50 and 100 g/kg GOS, for 30 days	Parallel design; control diet was follow-up infant formula; all other treatments were infant formula plus probiotic and/or prebiotic; 8 diets: control, probiotic (bifidobacteria), prebiotic at 3 GOS intakes, and synbiotic (GOS + bifidobacteria at 3 GOS intakes)	Male Sprague Dawley rats (*n* = 54), age 3 weeks	Apparent mineral absorption and retention (Ca, Mg and P) at 8–10, 18–20 and 28–30 days	Infant formulas supplemented with 100 g/kg GOS and 50 and 100 g/kg synbiotic treatments were most effective at increasing Ca, Mg and P bioavailability compared to control; apparent absorption and retention of Ca, Mg and P were initially above 80% but decreased at later measurement time points	Pérez-Conesa D, et al. 2006 (Ref. 57)
GOS, *Bifidobacteria bifidum and longum*	0, 12, 50 and 100 g/kg GOS, for 30 days	Parallel design; control diet was follow-up infant formula; all other treatments were infant formula plus probiotic and/or prebiotic; 8 diets: control, probiotic (bifidobacteria), prebiotic at 3 GOS intakes, and synbiotic (GOS + bifidobacteria) at 3 GOS intakes	Male Sprague Dawley rats (*n* = 54), age 3 weeks	Ca, Mg and P content of femur and tibia; cecal/colon morphology and pH	Femur and tibia Ca and P were improved most with prebiotic and synbiotic treatments; femur Mg content was reduced with all treatments relative to control while tibia Mg was unaffected; cecal wall weight was greater and pH lower with 100 g/kg GOS and synbiotic diets compared to control; synbiotic diets decreased colonic pH relative to control; colonic pH was negatively correlated with proximal and distal colon crypt depth and with distal colon cell density	Pérez-Conesa D, et al. 2007 (Ref. 58)
GOS, FOS	5.3% GOS–FOS mix, for 50 days	Parallel design; 4 diets: normal-Ca diet (AIN 93-G + 0.5% Ca), low-Ca diet (AIN 93-G + 0.3% Ca), normal-Ca diet + 5.3% GOS + FOS, or low-Ca diet + 5.3% GOS + FOS; GOS and FOS were fed in a 9:1 ratio	Male Wistar weanling rats (*n* = 32)	Apparent Ca absorption; BMD and BMC by DXA; tibia histomorphometry; fecal lactobacillus colony assessment; cecal morphology and pH; femur biomechanical analysis	Femur Ca and P content was significantly higher among GOS + FOS groups; GOS + FOS groups resulted in greater tibia length than other diets; lactobacillus colonies were also increased with GOS + FOS; GOS + FOS groups had greater cecum weight and lower cecal pH	Bryk G, et al. 2015 (Ref. 53)
Inulin	0, 3.75 and 7.5% inulin, for 3 weeks	Parallel design, SI: 4 age groups with control and treatment groups at each age; control diets included AIN 1993 mineral and vitamin mix; first 4 days at 3.5% inulin followed by 7.5% inulin	Male Wistar rats (*n* = 80), age 2.5, 5, 10, and 20 months	Fractional Ca and Mg absorption; cecal wall and content weights, pH and SCFA content	Inulin increased Ca and Mg absorption at all age groups but 10- and 20-month-old rats absorbed less Ca and Mg than younger rats; Ca and Mg retention decreased with age; cecal wall and content weights increased with a decrease in cecal pH at all inulin intakes; inulin also increased individual and total SCFAs relative to control	Coudray C, et al. 2005 (Ref. 100)
Inulin	0 and 10% inulin, for 40 days	Parallel design; run-in on semi-purified diets until 10 weeks of age; 6 diets: 0% inulin + 0.25% Ca, 0% inulin + 0.5% Ca, 0% inulin + 0.75% Ca, 10% inulin + 0.25% Ca, 10% inulin + 0.5% Ca, 10% inulin + 0.75% Ca	Male Wistar rats (*n* = 60)	Apparent Ca and Mg absorption at days 13 and 36; cecal pH and SCFA content	Ca and Mg absorption increased with all inulin treatments; inulin effects on Ca absorption were greatest with low and high-Ca diets in the short-term but persisted only for the low-Ca diet in the long-term; inulin also lowered cecal pH and increased cecal weight; inulin improved cecal SCFA content and total SCFA was greatest in the 10% inulin + 0.25% Ca diet compared to 10% inulin + 0.75% Ca	Coudray C, et al. 2005 (Ref. 91)
Inulin	3.75 and 7.5% inulin, for 26 days	Parallel design, SI; 8 study groups by age category and treatment group; first 4 days was 3.75% inulin followed by 7.5% inulin	Male Wistar rats (*n* = 80), age 2, 5, 10 and 20 months	Zn and Cu absorption; cecal morphology, SCFA content and pH determined	7.5% inulin increased cecal wall and content weights, and decreased cecal pH; while Zn and Cu excretion increased and retention decreased with age, inulin increased Zn and Cu retention; Zn absorption correlated with cecal acetate, propionate and total SCFA; bone Zn increased with increasing age but was not affected by inulin intake; bone Cu did not vary by inulin intake or age	Coudray C, et al. 2006 (Ref. 104)
Inulin	7.5% inulin, for 3 months	Parallel design; control diet designed to reflect “western diet”; 2 week run-in for dietary adaptation; measures at 1 and 3 months post-inulin diets; 4 diets: control, native inulin, reformulated inulin (combination of short- and long-chain fructans), dehydrated chicory	Male Wistar rats (*n* = 40), age 3 months	Apparent Ca and Mg absorption; BMD and BMC by DXA; bone biomarkers; femur biomechanical properties; cecal morphology, pH and SCFA content	Ca absorption increased with inulin at 1 month but the effect disappeared by 3 months; Mg absorption was improved at both 1 and 3 months; BMC was increased only with chicory inulin; BMD of diaphysis was improved by the chicory treatment but all other measures of bone strength or turnover were not different; inulin diets improved SCFA production with the chicory diet increasing propionate more than other inulin treatments	Demigné C, et al. 2008 (Ref. 114)
Inulin	7% inulin solution (v/w)	Parallel design; 4 diets: gluten-free diets with either the appropriate Ca content or reduced Ca content, or gluten-free diets with adequate or reduced Ca plus inulin	Male Wistar rats (*n* = 32), age 4 weeks	Ca, Mg, and P balance; fecal SCFA concentration; microbial community analysis by PCR-DGGE and quantitative rt-qPCR of 16S rRNA gene	Diets with inulin resulted in increased mineral absorption; Ca content of the diet influenced Ca, P, and Mg absorption; inulin stimulated fecal bifidobacteria content when dietary Ca was adequate; SCFA content in cecum was influenced by interaction between dietary Ca and inulin	Krupa-Kozak U, et al. 2017 (Ref. 92)
inulin	0, 5, 10 and 20% inulin, for 21 days	Parallel design	Male Wistar rats (*n* = 24), age 6 week	Apparent Ca absorption, cecal pools of Ca, P and Mg; cecal wall and content weights, pH and SCFA content	Inulin increased the cecal pool of Ca and P and to a lesser degree Mg; absorption of Ca was improved in a dose-dependent manner; total SCFA increased with inulin treatment; propionate content was increased with 10% inulin treatment relative to control	Levrat M, et al. 1991 (Ref. 99)
Inulin	10% inulin, for 15 days	Parallel design; rats were stratified based on body weight; run-in diet for 7 days prior to treatments which were modified from AIN-93G diet; 4 diets: 15% soy bean oil (SO), 15% Soy bean oil + EPA + DHA (FSO), 15% SO + 10% Synergy1^®^ or 15% FSO + 10% Synergy1^®^	Male Wistar rats (*n* = 24), age 6 week	Apparent mineral absorption and balance for Ca, Mg, Cu, Fe and Zn; bone mineral and biomechanical assessments (bone breaking); tibia and femur Ca and Zn content; tibia strength measures; cecal morphology and pH	Inulin improved apparent mineral absorption of Ca, Mg, Cu, Fe and Zn but FO and SO appeared to differentially influence absorption; FO negatively affected Mg absorption, while FSO potentiated the effects of inulin for Ca absorption and balance, and Cu, Fe and Zn absorption; bone Ca and Zn content were enhanced most by FSO + inulin; compared with controls, inulin increased cecal weight, number of crypts and cell density, and decreased cecal content pH	Lobo A, et al. 2009 (Ref. 111)
Inulin	10% inulin, for 22 days	Various parallel design experiments following animal feeding: Using chamber experiments for Ca flux following treatment with varying Ca doses, SCFA, intestinal pH and inulin exposures; Synergy1^®^ as inulin type	Male Sprague Dawley rats (*n* = 48)	Ca flux across excised intestinal tissue; morphological features of cecal and colonic tissue	Inulin increased Ca absorption in cecum; inulin altered gene expression of calbindin D9k, TRPV6 and Na–Ca co-transporters; inulin lowered cecal pH and increased cecal and colon content and wall weights; inulin increased intestinal SCFA content; pH alone had no influence on Ca absorption	Raschka L, et al. 2005 (Ref. 79)
Inulin	0, 5, or 10 g inulin/100 g of diet, for 22 weeks	Parallel design; Inulin diets in combination with 0.2, 0.5, 1.0 g Ca/100 g of diet	Male Wistar rats (*n* = 108), age 4 weeks	Whole-body BMC and BMD by DXA at 0, 10, 14, 18 and 22 weeks	5 g/100 g inulin had the greatest impact on whole-body BMC and BMD compared to control; BMC and BMD were improved by inulin at all Ca and age levels; bone area was differentially influenced by age and Ca	Roberfroid M, et al. 2002 (Ref. 96)
Inulin and polydextrose	5% inulin or polydextrose, for 4-weeks	Parallel design, SI; 2 month stabilization period with non-purified diet post-OVX; 6 groups: OVX + control diet (AIN-93M), sham + control diet, OVX + biweekly estradiol injections, remaining 3 groups were 5% inulin from Synergy1^®^ (SYN), 5% inulin from Fruitafit HD^®^ (FRT), or 5% polydextrose (PDX)	Female Sprague Dawley rats, age 5 months	Mineral content of diet, fecal and urine samples; BMD by DXA	Among OVX groups there were no differences in BMD due to fiber treatment; OVX + SYN, FRT, or PDX increased net Mg absorption and retention; OVX + PDX group showed a significant increase in Ca absorption and utilization; OVX + SYN group resulted in an increase in cecal wall weight, SCFA production and chronic Ca absorption; OVX + FRT group demonstrated higher cecal wall weight than SHAM-CON but not OVX-CON	Legette L, et al. 2012 (Ref. 97)
Inulin and soy isoflavones (IF)	0 mg/g inulin, 8 mg/g IF, or 50 mg/g + 8 mg/g inulin and IF, for 21 days	Parallel design; SI; control diet was AIN-93G; inulin and IF replaced cornstarch in diets	Male Sprague Dawley rats (*n* = 48), age 6 weeks	Ca absorption; femoral Ca content	Ca absorption and femoral Ca were significantly increased with IF compared to inulin; inulin + IF did not have an added benefit for Ca absorption or femoral content	Zafar T, et al. 2004 (Ref. 116)
Inulin, agave fructans	10% inulin, 10% agave fructan 1 (CAF1), 10% agave fructan 2 (CAF2), for 6 weeks	Parallel design with 5 groups: OVX or sham on control diet; remaining 3 OVX groups randomly assigned to supplemented diets; CAF1 and 2 derived from *Agave tequilana* with average DP of 22 and 13, respectively	Female C57BL/6J mice (*n* = 48), age 12 weeks	Apparent Ca and Mg absorption; bone biomarkers; femur microarchitecture; cecal and colon morphology; fecal SCFA content	Ca absorption but not Mg absorption increased with all fructan treatments at 3 and 6 weeks; Ca and Mg content of bone was improved on all fructan treatments; P content also increased relative to control treatments; osteocalcin increased with fructan treatment; cecal and colonic SCFA production did not differ	García-Vieyra M, et al. 2014 (Ref. 80)
Inulin, FOS	0 or 50 g/kg inulin + FOS mix, for 21 days	Parallel design, SI; control diet was AIN-93 with inulin + FOS replacing equal amounts of cornstarch and sucrose; run-in AIN-93 diet for 3 months following OVX	Female OVX Sprague Dawley rats (*n* = 26), age 6 months	True Ca absorption and kinetics; femur BMD and BMC by DXA	Inulin + FOS increased Ca absorption and retention compared to controls; inulin + FOS decreased bone turnover and improved bone Ca content; no differences were observed in bone-breaking strength	Zafar T, et al. 2004 (Ref. 105)
Inulin, soy isoflavone (genistein)	0 or 5% inulin, for 4 weeks	Parallel design, SI; 6 treatment groups: sham and OVX controls, OVX + daily estradiol injections, and remaining OVX groups with genistein, ITF or both; control diet was AIN-93M; 4 diets: control, 200 ppm genistein, 5% Synergy1^®^, 200 ppm genistein + 5% Synergy1^®^	Female OVX Sprague Dawley rats (*n* = 96), age 5 months	Ca balance and kinetics; femur and tibia BMD by underwater weighing; bone pQCT; femur and tibia biomechanical properties and Ca content; cecal SCFA content	Inulin improved Ca absorption; genistein improved total and trabecular BMD of the distal femur; rats consuming genistein + inulin experienced greater bone formation and resorption rates	Legette L, et al. 2011 (Ref. 118)
Lactose	0 or 10% lactose, for 6 weeks	Parallel design; 4 diets: vit-D-deficient; vit-D-deficient + 0.5% Ca + 0.3% P, vit-D-deficient + 1.5% Ca + 0.9% P + 15% lactose, and normal diet containing vit-D. 0.5% Ca + 0.3% P	Male Wistar rats (*n* = 26) resulting from in utero vitamin D deficiency, age 4 weeks	Plasma Ca and P and urine Ca at 3 and 6 weeks; femur and tibia histomorphometry and urine hydroxyproline at 6 weeks	Addition of lactose to vit-D-deficient diets improved femur and tibia measures relative to other vit-D-deficient groups but improvements did not reach those observed in the control group with adequate vit-D	Schaafsma G, et al. 1988 (Ref. 28)
Lactose, lactulose, xylitol, lactobionate, arabinose, raffinose, pyroglutamate, sorbitol, gluconate, and raftilose	2.5–15% prebiotic by weight of diet	Parallel design, DI; prebiotic treatments each tested over a single night incorporated into the standard diet (AIN-76A); treatments provided in various experimental iterations	Male Fischer 344 rats (*n* = 8–9 rats per group), age 5–38 weeks	Fractional Ca absorption	All sugars increased fractional Ca absorption relative to control diets lacking prebiotics; additive effects of vit-D and lactulose together were observed; fractional Ca absorption following lactulose consumption and cecectomy remained high	Brommage R, et al. 1993 (Ref. 36)
Oligofructose	0, 25, 50 and 100 g/kg oligofructose, for 16 weeks	Parallel design; 7 groups including sham and OVX control groups; weanling rats were placed on diets with 8 g/kg Ca and 5 g/kg P as run-in until 5 months of age; 6 diets: control (5 g/kg Ca + 0 g/kg oligofructose), 5 g/kg Ca + 25 g/kg oligofructose, 5 g/kg Ca + 50 g/kg oligofructose, 5 g/kg Ca + 100 g/kg oligofructose, 10 g/kg Ca + 0 g/kg oligofructose, 10 g/kg Ca + 50 g/kg oligofructose	Female OVX Fisher 344 rats (*n* = 96), age 5 months	Apparent Ca absorption; bone microarchitecture	Oligofructose was most effective at influencing Ca absorption and bone microarchitecture when dietary Ca was high; bone loss due to OVX was prevented but different Ca and oligofructose combinations resulted in different microarchitecture changes; compared to controls, 5 g/kg Ca + 100 g/kg oligofructose and 10 g/kg Ca + 50 g/kg oligofructose improved femur Ca content while only 10 g/kg Ca + 50 g/kg oligofructose increased lumbar Ca content; 5 g/kg Ca + 25 g/kg and 100 g/kg oligofructose increased trabecular bone area compared to controls, as did both 10 g/kg Ca diets; at 10 g/kg Ca trabecular number increased with cortical thickness reaching higher levels when 50 g/kg oligofructose was added to the diet	Scholz-Ahrens K, et al. 2002 (Ref. 108)
Oligofructose, inulin	0 or 10% oligofructose or inulin, for 28 days	Parallel design; diet intervention followed a 21 day adaptation period; diets contained 5400 and 520 mg/kg of Ca and Mg, respectively; 5 diets: control, 10% oligofructose (OF), 10% HP-inulin, 10% Synergy1^®^, or 10% branched-chain inulin	Male Wistar rats (*n* = 50). age 6 weeks	Ca and Mg absorption; cecal wall and content weight, pH and SCFA content	Ca balance was increased among all ITF treatments relative to controls but no differences were observed between ITF groups; Mg balance was improved with all ITF treatments compared to control; oligofructose and Synergy1^®^ increased Mg absorption more than other ITF treatments; cecal pH was lower and cecal wall and content weight were increased with all ITF treatments; SCFA content was increased among all ITF groups relative to control	Coudray C, et al. 2003 (Ref. 98)
Oligofructose + acacia gum, *Lactobacillus acidophilus NCC90*	2.5% oligofructose + acacia gum, for 16 weeks	Parallel design; 5 groups: sham-operated and OVX controls consumed semi-purified diet with 0.7% Ca and 0.5% P (SP diet), remaining 3 groups consumed SP diet with lactobacillus (probiotic), SP diet with 2.5% oligofructose + acacia gum (prebiotic), and SP diet with probiotic and prebiotic (synbiotic); 2 week run-in on SP diet; prebiotic replaced cornstarch in diets	Female OVX Fisher-344 rats (*n* = 80), age 21 weeks	Apparent absorption and retention of Ca and P; bone biomarkers; bone microarchitecture; intestinal weight and pH; fecal microbial culture analyses	Prebiotic increased Ca absorption, decreased urinary phosphorus, increased cecal content weight, lowered cecal and colon pH and tended to increase calcium balance compared to control; synbiotic reduced pH in small intestine and cecum, and stimulated colonic absorption surface area; synbiotic prevented OVX-induced Ca and P losses in lumbar vertebrae; synbiotic bone-sparing effects were associated with greater fecal bifidobacteria at 6 weeks and greater Bacteroides at 16 weeks; BAP had a tendency to decrease with prebiotic and synbiotic treatments and BAP was negatively correlated with femur P and lumbar Ca and P content	Scholz-Ahrens K, et al. 2016 (Ref. 75)
Resistant starch (RS), daidzein (DZ)	4 diets: control, 0.1% DZ supplement, 12% RS supplement, 0.1% DZ + 12% RS, for 40 days	Parallel design; 6 groups: OVX or sham controls on AIN-93G with corn oil instead of soybean oil; 3 OVX groups consumed supplemented diets; final group was OVX + daily subcutaneous administration of 17 β-estradiol; 4 day run-in on AIN-93G; daidzein/RS replaced sugar/cornstarch in diet	Female ddY mice (*n* = 36), age 8 weeks	Tibia BMD; amplification of the fecal bacteria 16S DNA and terminal restriction fragment length polymorphism	RS alone did not improve BMD; BMD in the DZ + RS group tended to be higher than in the DZ group; RS feeding resulted in a greater proportion of bifidobacteria species	Tousen Y, et al. 2011 (Ref. 76)
Resistant starch (RS), soy isoflavones (IF)	4 diets: control, 0.05% IF, 9% RS, and 0.05% IF + 9% RS, for 42 days	Parallel design; 5 groups: OVX or sham controls on AIN-93G with corn oil instead of soybean oil; 3 groups consumed supplemented diets; 4 day run-in on AIN-93G; IF/RS replaced sugar/cornstarch in diet; animals pair-fed	Female ddY mice (*n* = 35), age 8 weeks	Femur BMD and trabecular microarchitecture; cecal content weight and pH; amplification of the fecal bacteria 16S DNA and terminal restriction fragment length polymorphism; bone marrow inflammation	IF + RS increased equol production, prevented the OVX-induced decline in trabecular BMD of the distal femur and improved inflammation in bone marrow; no difference in bone measures between IF + RS and IF diets; diets with RS increased cecal bifidobacteria content	Tousen Y, et al. 2016 (Ref. 77)
Resistant Starch (RS60 and 75), Soluble Corn Fiber (SCF), Soluble Fiber Dextrin (SFD), Pullulan, Polydextrose (PDX), Inulin, and Synergy1^®^ (Inulin + FOS)	4% of prebiotic by weight of diet, for 12 weeks	Parallel design, SI; initially 2 weeks of diets were AIN-93G + 10% prebiotic but due to loose stools in several groups, the % fiber was reduced to 5% after 2 weeks for all treatments and again after another 3 weeks in SCF, SFD, and PDX groups to 4% prebiotic and 1% cellulose; prebiotics replaced cornstarch in diets	Male Sprague Dawley rats (*n* = 150), age 4 weeks	Ca absorption and mineral (Ca, Zn, Fe, Mg, K and Cu) retention; whole-body BMD, BMC and body composition by DXA; bone density (by water displacement), pQCT and bone-breaking strength of femurs; cecal morphology and fecal SCFA concentrations	Whole-body BMD and BMC were greatest following SCF and SFD consumption; Ca and Mg concentrations in bone and femur contents were improved by RS60, RS75, SFD, PDX, inulin and inulin/FOS; Cu retention was improved by all fibers except inulin; Zn femur content was improved by all fibers but pullulan and SCF; Fe retention was improved by SCF; cecal content weight improved with SFD, SCF, PDX, inulin and Synergy1^®^; all fibers except RS60 and RS75 increased total SCFA and propionate in the cecum; peak breaking force of femurs was significantly improved by SCF and SFD	Weaver C, et al. 2010 (Ref. 41)
sc-FOS	0 or 100 g/kg of sc-FOS, for 10 days	Parallel design; adequate Ca diet (0.5%) or low Ca diet (0.01%) were administered in conjunction with both sc-FOS treatments	Male Sprague Dawley rats (*n* = 20), age 4 weeks	Serum Ca concentration; calbindin D9k protein expression; circulating markers of vitamin D and Ca metabolism; small intestine and cecum weight	sc-FOS increased calbindin D9k expression in the large intestine; changing the Ca level of the diet did not have an effect on protein expression; positive correlations were observed between 1,25(OH)_2_D and calbindin D9k; cecum and colon tissue weight were significantly increased with the addition of sc-FOS; serum Ca was unaffected by sc-FOS	Takasaki M, et al. 2000 (Ref. 102)

*1,25(OH)*_2_ *D* 1,25 dihydroxy vitamin D, *BMC* bone mineral content, *BMD* bone mineral density, *BMI* body mass index, *Ca* calcium, *CTx* C-telopeptide of type I collagen, *Cu* copper, *DI* double-isotope method, *DNA* deoxyribose nucleic acid, *DXA* dual-energy X-ray absorptiometry, *Fe* iron, *FOS* fructooligosaccharides, *GOS* galactooligosaccharides, *IF* isoflavones, *ITF* inulin-type fructans, *Mg* magnesium, *OVX* ovariectomized, *P* phosphorus, *PCR-DGGE* polymerase chain reaction-density gradient gel electrophoresis, *PDX* polydextrose, *rRNA* ribosomal ribonucleic acid, *RS* resistant starch, *rt-qPCR* real-time quantitative polymerase chain reaction, *sc-FOS* short-chain fructooligosaccharides, *SCFA(s)* short-chain fatty acid(s), *SI* single-isotope method, *vit-D* vitamin D, *Zn* zinc

### In Vitro Calcium Availability and Absorption Models

Of the data currently available, findings from in vitro cell models suggest that prebiotics foster cellular calcium uptake but that microbes differentially regulate this process. Caco-2 cell monolayer studies indicate that difructose anhydride III, difructose anhydride IV, FOS, and raffinose (trisaccharide made of galactose, glucose, and fructose) all increased paracellular calcium absorption in a dose-dependent manner [93]. Conversely, in a similar Caco-2 cell model, inoculation with a human intestinal bacterial suspension resulted in a decreased presence of calcium in the basolateral filtrate but greater intracellular calcium content [94]. This suggests that prebiotics in the presence of gut microbiota influenced luminal uptake of calcium while possibly limiting further absorption into the blood. It is possible that specific members of the intestinal microbial community are responsible for facilitating calcium absorption. In the latter study, Caco-2 cells were only inoculated with Lactobacillus, Enterobacteriaceae, and Enterococcus after extraction from human feces [94]. Previously, a comparison of two probiotic strains (*Lactobacillus salivarius* and *Bifidobacterium infantis*) with commensal *E. coli* suggested that *Lactobacillus salivarius* significantly increased cellular calcium uptake, while E. coli slightly but non-significantly increased calcium uptake in comparison to *B. infantis* which had no effect [95].

### Animal Models—Prebiotic Effects at the Intestinal Level

The majority of animal data exist for fructose-based prebiotics, primarily inulin and FOS [75, 80, 96], but positive impacts of GOS [24] and polydextrose [97] on calcium absorption have also been observed. Because animal studies are more cost and time effective, they have been instrumental for understanding the effects of varying prebiotic doses, types, and chain lengths (DP) on mineral absorption [98, 99]. Despite a diverse body of literature regarding dose and prebiotic structural components, the animal literature is supportive; however, greater evidence is needed to understand how age, treatment duration, and microbial community constituents influence mineral absorption at the intestinal level.

#### Effects of Age

Intestinal physiology changes across the lifespan to accommodate different metabolic needs. Therefore, age may play an important role in facilitating prebiotic-induced mineral absorption in the intestine. To date, few studies have compared prebiotic effects across a wide age range. A well-designed study evaluating the effects of inulin consumption at 3.75% of diet weight for four days followed by 3 weeks with 7.5% inulin content reported greater calcium and magnesium absorption in rats aged 2, 5, 10, and 20 months when compared to non-inulin-fed controls [100]. The magnitude increase for magnesium was more similar across all ages, while inulin-induced calcium absorption was significantly greater among 2- and 5-month-old rats (~ 66%) compared to older rats (~ 27%). Intestinal pH, cecal wall and content weights, and fecal SCFA concentrations were also evaluated. Intestinal morphology and pH were modified by inulin but the effects did not differ by age; however, an age–inulin interaction emerged for SCFA content with greater acetate and butyrate in 10-month-old rats compared to the other three age groups [100]. A potential explanation for the blunted effect of inulin on calcium absorption in the older animals may be the tight regulation of calcium flux compared to that for magnesium. A reduction in calcium transporters involved in active transport in the small intestine, but not the large intestine, has been observed with inulin consumption [101, 102]. Further, calcium absorption effects have been observed to decline slightly throughout prebiotic experimental trials suggestive of treatment adaptation [103]. Similar findings have been reported for intestinal zinc and copper absorption following inulin consumption. Despite increased absorption with 7.5% inulin across age groups, inulin-induced zinc and copper absorption was lower in older rats (11 and 21 weeks) compared to younger animals (3 and 6 weeks) [104]. This presents important questions of whether prebiotic effects can be sustained with long-term consumption and if varying prebiotic approaches are needed at different life stages.

In conjunction with age, prebiotics may also elicit positive effects on calcium absorption in the context of estrogen deficiency. Calcium absorption was significantly improved in ovariectomized rats fed 5% GOS for 30 days but the benefit disappeared after 20 days of GOS supplementation [24]. This effect was accompanied by significant increases in cecal wall and content weight, and greater luminal contents of acetic, propionic, butyric, and succinic acids as well as total SCFA content. Similar effects were observed among ovariectomized rats consuming inulin-enriched diets (5% of diet) for 21 days which resulted in improved calcium balance [105]. Prebiotics have also been shown to elicit benefits in the presence of nutritional deficiencies in the context of estrogen deficiency. Four-week consumption of the prebiotic disaccharide, difructose anhydride III (DFAIII; 15 g/kg diet), increased calcium absorption in vitamin D-deficient ovariectomized rats [106]. Taken together, findings from animal models suggest that mineral absorption may differ by age and estrogen deficiency. For example, when given similar doses of FOS (50 g/kg) a study in young growing rats found improvements in calcium absorption [107], while another study in ovariectomized rats did not observe an effect on calcium absorption [108]. In the ovariectomized rats, benefits to calcium absorption and retention were most consistently observed over 16 weeks when high calcium intakes (10 g/kg) were combined with 50 g/kg oligofructose, while phosphorus retention was greater with 100 g/kg oligofructose through 4 weeks and 50 g/kg oligofructose through 8 weeks [108]. This ultimately suggests the importance of hormonal status and a need to understand how similar prebiotic doses influence different minerals at different stages of life, which doses are most effective, and whether benefits persist across longer interventions.

#### Prebiotic Type, Dose, and Duration Effects

The effects of prebiotics may be dependent upon the type of prebiotic used, and the dose and length of treatment. Following an elegant evaluation of nine different fibers (two resistant starches, soluble corn fiber, soluble fiber dextrin, pullulan, polydextrose, inulin, long-chain inulin + short-chain FOS, and cellulose), calcium and iron retention, as measured by balance studies, were unaffected by all treatments despite beneficial effects on bone which are discussed in the next section relating to bone outcomes [41]. Magnesium retention was only affected by soluble fiber dextrin, while copper retention was improved by all fibers except inulin, and zinc retention was improved by soluble fiber dextrin, both resistant starches, and the inulin–FOS mixture [41].

Varying the dose of selected prebiotics in animal models appears to yield similar results with regard to calcium absorption [36, 109, 110]; however, chain length and branching of the prebiotic may have a greater impact on mineral bioavailability. Adult male rats consuming different fructan combinations that contained a range of chain lengths and branching found that only an oligofructose–inulin combination resulted in significantly increased calcium absorption [98]. The combination of short- and long-chain fructans in this study may have resulted in synergistic effects by allowing for prolonged fermentation and absorption of calcium throughout the large intestine.

The dose of calcium administered may also interact to influence prebiotic effectiveness, especially in states of estrogen deficiency. Oligofructose proved more effective at increasing calcium absorption and retention in ovariectomized rats when consuming a high-calcium diet, while phosphorus absorption decreased relative to the control treatment at this intake of calcium [108]. The influence of calcium dose was also apparent when growing rats consumed 10% inulin for 40 days in conjunction with calcium intakes at 0.25, 0.50, and 0.75% by weight of the diet [91]. Findings from this study suggested that, during periods of growth, inulin initially increased calcium absorption at all calcium concentrations but dissipated with the 0.75% calcium diet by the end of the study. Overall, the effect of prebiotics on calcium absorption can be seen in a few days but which types of prebiotics and respective doses are most appropriate requires further exploration.

### Animal Models—Prebiotic Effects at the Level of Bone

Due to their accelerated lifespans, rodent models have proven highly useful for studying the effects of prebiotics on bone mineral density, skeletal tissue structure/geometry, and fracture risk. Despite this fact, fewer studies have evaluated the impact of prebiotics on measures of skeletal health. Broadly, prebiotics have been shown to increase the mineral content [26, 111, 112], architecture, and strength [111, 113–115] of bone. Discrepancies have emerged such that findings have not been consistent among animal models meant to depict periods of juvenile skeletal growth [98, 116]. Occasional findings have also been suggestive of improved bone architecture and strength when bone mineral density was not affected [113, 114]. Among studies that report benefits to bone tissue, it has been postulated that beneficial effects are the result of changes in the more metabolically active, trabecular bone.

#### Effects of Age

Growing animals experience improvements in bone mineral content and density [96] and trabecular bone volume of the femur [112] with FOS consumption. Similarly, GOS at varying intakes improved volumetric bone mineral density and breaking strength of both the femur and tibia [26]. In postmenopausal animal models, FOS may be more effective at eliciting benefits to bone when combined with other ingredients, or as synbiotics. FOS alone has resulted in positive [80] and null effects [97] on bone mineral density. However, administering inulin and FOS reduced bone resorption, increased femoral calcium content, and improved bone mineral density [105]. Further, combining FOS and soy isoflavones improved bone mass of the femur in ovariectomized rats in one study [117], while no synergistic effect was observed in another [118].

#### Prebiotic Type, Dose, and Duration Effects

In a 12-week study comparing 8 different prebiotics to cellulose in growing rats, femur calcium uptake was increased significantly by the inulin–FOS mixture, while measures of bone strength (peak breaking force and cortical area and thickness) were most improved by soluble corn fiber and soluble fiber dextrin [41]. This study attempted to correlate findings with mechanistic data by evaluating changes in fecal SCFA concentrations and cecal morphology. While the production of SCFAs was slightly associated with bone benefits, overall, mechanistic findings were unclear as different calcium and bone parameters were associated with differing intermediate intestinal variables.

In a similarly complex 16-week study of ovariectomized rats that compared varying levels of dietary calcium (5 or 10 g/kg) and oligofructose (25, 50 and 100 g/kg), differential effects on bone were also observed [108]. Across the 16 weeks, both femur calcium and phosphorus content decreased but at recommended calcium intakes (5 g/kg), 25 g/kg of oligofructose preserved trabecular area and thickness, 50 g/kg increased trabecular perimeter, and 100 g/kg improved trabecular perimeter and trabecular area. When calcium intake increased to 10 g/kg, the addition of 50 g/kg of oligofructose resulted in increased calcium content of the lumbar vertebrae and greater trabecular area and thickness but no change in trabecular number when compared to a similar calcium diet with no oligofructose. The authors of this study concluded that ovariectomy-induced bone loss was prevented but that calcium and oligofructose interacted to affect the geometry and architecture of bone. Interestingly, oligofructose elicited the strongest effects on bone with 10 g/kg of calcium, as it was the only combination that significantly maintained trabecular connectivity while increasing cortical bone thickness. In comparison to another study which varied dietary calcium content (0.25, 0.50, 0.75%) with and without inulin for 40 days, no effect of inulin was observed for either calcium or magnesium content of the tibia despite increases in intestinal mineral absorption [91]. However, among growing male rats followed for 22 weeks, whole-body bone mineral content and area were improved by the addition of inulin-type fructans (5 and 10 g/100 g diet) regardless of dietary calcium content (0.2, 0.5, and 1.0 g/100 g diet) [96]. The effect on bone was not improved further by increasing prebiotic consumption to 10 g/100 g. While findings from the former study in ovariectomized rats suggest varying effects of prebiotic and calcium doses over time, the findings from the latter study in growing rats imply that prebiotics benefit bone regardless of dietary calcium content. Currently, few studies have followed animal models for long periods of time which make it difficult to understand how differential prebiotic doses in combination with varied calcium intakes influence bone properties across life. More work is needed to understand these combined effects.

As suggested earlier, prebiotic structure (branching and chain length) may also influence calcium and bone health outcomes. In a study comparing agave fructans (similar degrees of polymerization but one branched and the other linear chains) with inulin, the agave fructans were slightly more effective at preserving structural properties of trabecular bone in ovariectomized rats [80]. Further, the more branched of the two agave fructans (CAF1), which also had a greater DP, resulted in slightly higher femoral calcium content. All of the fructan treatments in this study resulted in significantly greater intestinal SCFA content and greater circulating concentrations of osteocalcin. This particular study highlights that bone may be influenced differently by prebiotics within the same class, suggesting that specific FOS can be used to correct different structural features of weight-bearing bones.

### Animal Models—Influence of Gut Microbes on Prebiotic-Induced Bone Effects

Very few studies have evaluated the role of gut microbiota in prebiotic–bone interactions. In a dose–response study, rats consuming GOS had greater intestinal microbial community diversity and improved bifidobacteria content after 8 weeks [26]. Consumption of GOS also led to greater calcium and magnesium absorption and improved femur and tibia strength. In this study, changes to intestinal tissue (greater cecal wall and content weight, and decreased luminal pH) and microbial community structure were correlated with calcium absorption and femur bone mineral density outcomes. These findings suggest the importance of gut microbes as a potential mechanism for prebiotic benefits to bone.

The role of microbial community members was further supported by findings from a comparison of prebiotics, probiotics, and synbiotics during a period of estrogen deficiency. In this investigation, ovariectomized rats consumed either *Lactobacillus acidophilus* (PRO), oligofructose + acacia gum (PRE), or combined *L. acidophilus* with oligofructose + acacia gum (SYN) for 16 weeks [75]. While PRE was the only treatment to significantly improve calcium absorption, no treatment resulted in improved femoral calcium content relative to control. SYN consumption resulted in significant improvements in lumbar vertebrae calcium content and a slight reduction in bone alkaline phosphatase. Although none of the treatments improved measures of bone histomorphology, the SYN-induced changes in lumbar vertebrae were associated with greater bifidobacteria counts at 6 weeks and greater *Bacteroides* at 16 weeks. Further, PRE and SYN reduced the pH of intestinal contents, specifically in the cecum. PRE tended to increase the weight of cecal contents, while SYN increased colonic tissue weight. As a whole, these data suggest that synbiotics may prevent bone loss by increasing fermentation capacity, microbial mass, and surface area in the intestine while also reducing bone turnover.

## Evidence from Clinical Trials

While the gross majority of human research indicates that prebiotics are beneficial for improving calcium absorption, fewer studies have evaluated their effects on bone mineral density. Even fewer studies have incorporated gut microbial measures as a potential mechanistic outcome for positive calcium metabolism effects. This section summarizes the current literature while taking into consideration conflicting results (Table 2). Variation in treatment conditions, intervention duration, lack of controlled diets, host genetics, baseline bone mineral status, and participant age will be addressed.

**Table 2:** A summary of prebiotic interventions among humans to evaluate their effectiveness and efficacy in altering mineral absorption, bone health outcomes, and intestinal parameters

Prebiotic/substance	Treatment dose and duration	Study design	Human population description	Measures/analyses	Findings	Abbreviated reference
FOS	8 g/day FOS or maltodextrin-sucrose (control), for 3 months	Randomized, double-blind parallel design; 2 daily doses of 200 mL of tap water + 4 g FOS or maltodextrin-sucrose	Korean postmenopausal women (*n* = 26), mean age 60 years and ~ 12 years since menopause	Apparent Ca, P, Fe and Zn absorption; BMD by DXA; bone biomarkers	FOS increased apparent absorption of Ca and Fe but not P and Zn; urinary Ca excretion was not different between groups; serum alkaline phosphatase decreased among FOS group compared to controls	Kim Y, et al. 2004 (Ref. 126)
FOS, lactose	0 or 4 g/day FOS in milk, for 12 weeks	Parallel design; milk consumed as 2 servings per day; 2 groups: Ca and vit-D fortified milk (1000–1200 mg/day Ca and 15 ug/day vit-D) + FOS (4 g/day) and regular milk (500 mg/day),	Premenopausal (*n* = 136, mean age 41 years) and postmenopausal (*n* = 121, mean age 59 years) women; Chinese	Bone biomarkers; vit-D status	Postmenopausal women: Ca and vit-D milk + FOS significantly reduced bone resorption (lower CTx) and improved vit-D status; premenopausal women: vit-D status improved but markers of bone turnover were unchanged	Kruger M, et al. 2016 (Ref. 61)
GOS	0 or 20 g/day of transgalactooligosaccharides via 200 mL of yogurt, for 9 days	Placebo-controlled crossover design, DI; sucrose was added to the control yogurt; gradual dose increase from 10 g/day to 20 g/day over 5 days; 19 day washout; Ca intake not reported	Women, 5 + years postmenopausal (*n* = 12), age 55–65 years	True Ca absorption	GOS increased true Ca absorption; no observable increase in urinary Ca excretion	van den Heuvel E, et al. 2000 (Ref. 27)
GOS	0, 5, or 10 g/day GOS, for 3 weeks	Randomized, double-blind, parallel design, DI; GOS consumed in two milk-based smoothies per day	Adolescent girls (*n* = 31), age 10–13 years	Fractional Ca absorption; gut microbial community by PCR-DGGE; bifidobacteria by rt-qPCR	5 g/day GOS group showed the greatest increase in Ca absorption; no dose–response effect observed on Ca absorption; DGGE profiles did not differ by treatment but bifidobacteria content was also increased with 5 g/day GOS	Whisner C, et al. 2013 (Ref. 121)
GOS, polydextrose (PDX)	4 g GOS–PDX mix/L of formula, for 2 weeks	Randomized, double-blind, parallel design, DI; non-prebiotic formula, prebiotic (GOS + PDX in a 1:1 ratio at 4 g/L), human milk + drops of vit A, C, and D	Infants (*n* = 74), age 56–70 days	Fractional Ca absorption; serum 25-OH vit-D	Human milk resulted in greater total fractional Ca absorption than both formulas; addition of prebiotic did not improve Ca absorption relative to non-prebiotic supplemented formula; Ca absorption efficiency was higher in human milk-fed group	Hicks P, et al. 2012 (Ref. 120)
Inulin	4 formulas with 0.0, 0.75, 1.00, or 1.25 g/day inulin, for 35 days	Randomized, parallel design; 7 day diet run-in, 14 day treatment followed by a 14 day washout period	Healthy, formula-fed infants from Malaysia (*n* = 36), age 5–12 months	Apparent Ca, Fe, Zn, Mg and Cu absorption; fecal SCFA	1 g/day inulin increased Fe absorption and retention; 0.75, 1, and 1.25 g/day inulin increased Mg absorption and retention; 0.75 g/day inulin improved Zn absorption and retention; Ca and Cu were unaffected by inulin; SCFAs were not influenced by inulin supplements	Yap K, et al. 2005 (Ref. 119)
Inulin	8 g/day of inulin Synergy1^®^ or sucrose (control), for 3 weeks	Placebo-controlled crossover design, DI; 2 week washout; treatments mixed with 8 oz. Ca-fortified orange juice in morning and evening; participants maintained on diets with 1200 mg/day Ca during study period	Girls, varied race/ethnicity (*n* = 54), age 10–15 years	Fractional Ca absorption	Ca absorption improved with Synergy1^®^; those with lower Ca absorption on placebo received the greatest benefit from Synergy1^®^	Griffin I, et al. 2003 (Ref. 51)
Inulin	10 g/day Synergy1^®^ or maltodextrin (control), for 6 weeks	Randomized, double-blind crossover design, DI; 6-week washout period; measures at 0, 3 and 6 weeks	Postmenopausal women (*n* = 15), ≥ 10 years past menopause; mean age 72 years	Fractional Ca and Mg absorption; BMD by DXA; bone biomarkers	Inulin increased Ca and Mg fractional absorption; urinary deoxypyridinoline cross-links and osteocalcin concentrations increased across the 6 weeks of inulin supplementation; two-thirds of the cohort responded positively to inulin with regard to mineral absorption and response was predicted by baseline lumbar BMD	Holloway L, et al. 2007 (Ref. 52)
Inulin	0 or 9 g/day of Synergy1^®^, for 3 weeks	Randomized, double-blind crossover design, SI; Ca-fortified cereal and 1500 mg/day Ca intakes; 2 week washout period	Adolescent girls (*n* = 14), age 11–13 years; mixed race/ethnicity cohort	Fractional Ca absorption and retention	No significant differences in Ca absorption and retention were observed between treatments	Martin B, et al. 2010 (Ref. 125)
Inulin	0 or 8 g/day of Synergy1^®^, for 12 months	Parallel design, DI; inulin or maltodextrin control mixed with 180–240 mL of Ca-fortified orange juice and consumed at breakfast	Girls and boys, varied race/ethnicity (*n* = 95), age 9–13 years	Fractional Ca absorption at baseline, 8 weeks and 12 months; DXA at baseline and 12 months; genotyping	Ca absorption was greater at 8 weeks and 12 months in inulin group; whole-body BMD and BMC were greater at 12 months; FF and Ff Foc1 genotypes had greater responses to inulin compared to control	Abrams S, et al. 2005 (Ref. 47)
Inulin	0 or 8 g/day of Synergy1^®^, for 12 months	Parallel design, DI; inulin or maltodextrin control mixed with 180–240 mL of Ca-fortified orange juice and consumed at breakfast	Girls and boys, varied race/ethnicity (*n* = 48), age 9–13 years	Fractional Ca absorption; urinary mineral excretion; responder (> 3% increase in Ca absorption) status	67% of subjects were classified as “responders;” responders at 8 weeks had greater BMC than non-responders at 12 months	Abrams S, et al. 2007a (Ref. 48)
Inulin	0 or 8 g/day of Synergy1^®^, for 8 weeks	Parallel design, DI; kinetic modeling performed on responders; inulin mixed with 120 mL Ca + vit-D fortified orange juice; participants maintained on diets with 800–1000 mg/day Ca during study period	Women and men, varied race/ethnicity (*n* = 13), age 18–27 years	Fractional Ca absorption and kinetic modeling; responder (> 3% increase in Ca absorption) status	Inulin increased Ca absorption in the colon, as measured in “responders,” with colonic absorption defined as > 7 h	Abrams S, et al. 2007b (Ref. 49)
ITF, lactose	1.75 g ITF/cup of fermented milk, for 2 weeks	Parallel design; matched for age, time after menopause, BMI and dietary calcium intake; 175 ml drink consumed at bedtime; mean habitual Ca intake of 906.4 ± 53.2 mg/day; 3 groups: fermented milk (f-milk), fermented milk supplemented with Ca (510 mg/cup) (f-milk + Ca), and fermented milk supplemented with Ca (510 mg/cup), ITF (1.75 g/cup) and caseinophosphopeptides (0.175 g/cup) (f-milk + Ca + ITF + CPP)	Women, 10.5 + years postmenopausal (*n* = 85), age 48–67 years	Bone biomarkers and hormones; serum minerals; urinary Ca and P	f-Milk, independent of Ca, ITF and CPP additions, decreased nighttime deoxypyridinoline excretion; urinary Ca and P increased during nights for the f-milk + Ca + IFT + CPP group which was attributed to greater intestinal absorption	Adolphi B, et al. 2009 (Ref. 59)
Lactose	3 groups: milk + lactose, milk lacking lactose + glucose, water + Ca (control), for 6–10 weeks	Randomized crossover design, DI; study treatments were administered to normal-lactase or lactase-deficient males	Otherwise healthy, lactase-sufficient and lactase-deficient males (*n* = 15), age 22–32 years	Fractional Ca absorption	Milk + lactose among lactase-deficient males increased Ca absorption; Ca absorption did not differ among lactase-deficient and sufficient males when consuming milk + glucose	Griessen M, et al. 1989 (ref 30)
Lactose	2 groups: kefir-fermented milk (1600 mg) + calcium bicarbonate (CaCO_3_, 1500 mg) or CaCO_3_ alone, for 6 months	Parallel design; included those with and without fracture	Women and men with osteoporosis (*n* = 40), age 64 + years	Bone biomarkers; BMD by DXA	Kefir with CaCO_3_ provided no additional benefit to Ca absorption; baseline bone turnover was most predictive of BMD changes at 6 months; kefir was a significant predictor of changes in BMD of the total hip	Tu M, et al. 2015 (Ref. 60)
Lactulose	0, 5, and 10 g/day of lactulose dissolved in 100 mL of water with benzoic acid, for 9 days	Dose–response, placebo-controlled crossover design, DI; aspartame used as placebo control; 19 day washout	Women, 5 + years postmenopausal (*n* = 12), age 56–64 years	Fractional Ca absorption	Ca absorption increased dose-dependently but difference between 5 and 10 g was not significant; Ca absorption was significantly higher with 10 g lactulose relative to control; Ca excretion did not differ by treatment	van den Heuvel E, et al. 1999a (Ref. 37)
Oligofructose	15 g/day of oligofructose or sucrose (control), for 9 days	Randomized, double-blind crossover design, DI; 19 day washout period; control and treatment were given in 100 mL of orange juice + standard breakfast containing 200 mg Ca	Healthy, male adolescents (*n* = 12), age 14–16 years	Fractional Ca absorption	Oligofructose increased fractional Ca absorption relative to control; no association was observed between Ca absorption and urinary Ca excretion	van den Heuvel E, et al. 1999b (Ref. 122)
Oligofructose and inulin	8 g/day of oligofructose, Synergy1^®^, oligofructose + Synergy1^®^,or sucrose (control), for 3 weeks	Placebo-controlled crossover design, DI; 2 week washout; treatments mixed with 8 oz. Ca-fortified orange juice in morning and evening; participants maintained on diets with 1200–1300 mg/day Ca during study period	Girls, varied race/ethnicity (*n* = 59), age 11–14 years	Fractional Ca absorption; urinary mineral excretion	Ca absorption was significantly higher in the Synergy1^®^ group; oligofructose consumption did not result in an improvement in Ca absorption relative to control	Griffin I, et al. 2002 (Ref. 50)
sc-FOS	0 or 3.6 g/day sc-FOS, for 24 months	Randomized, double-blind crossover design; interventions provided as chewable chocolate-flavored supplements; 2 supplements taken per day; 3 groups: 800 mg/day Ca, 800 mg/day Ca + 3.6 g/day sc-FOS, or 9 g/day of maltodextrin (control)	Postmenopausal women (*n* = 300), age 45–75 years; non-osteoporotic; ~ 12–13 years since menopause	BMD by DXA; bone biomarkers; measurements at 0, 12 and 24 months	Ca alone and Ca + sc-FOS did not result in smaller BMD losses compared to maltodextrin control; of women with osteopenia, declines in BMD were smaller among Ca + sc-FOS group compared to maltodextrin; Ca alone and Ca + sc-FOS groups experienced greater declines in C-telopeptides of type I collagen at 12 months compared to maltodextrin; Ca + sc-FOS resulted in greater decline in osteocalcin when compared to maltodextrin at 24 months	Slevin M, et al. 2014 (Ref. 127)
sc-FOS	10 g/day sc-FOS or sucrose (control), for 5 weeks	Randomized, double-blind crossover, design, SI; 3-week washout period; first 4 days 5 g/day sc-FOS at lunch, followed by 10 g/day sc-FOS delivered at lunch and dinner; first 23 d of study participants followed habitual diet, followed by controlled diets containing ~ 900 mg/day Ca, ~ 250 mg/day Mg and ~ 12 g/day fiber	Postmenopausal women (*n* = 12), > 2 years into menopause without hormone replacement; age 50–70 years	Fractional Ca absorption; bone biomarkers; vit-D metabolites and hormones	No significant difference in Ca absorption on sc-FOS vs. control; slightly higher Ca absorption observed in women > 6 years postmenopausal; urinary Ca excretion and bone turnover markers did not differ by supplement group; 1,25(OH)_2_D decreased slightly with sc-FOS relative to control but effect was not significant	Tahiri M, et al. 2003 (Ref. 123)
sc-FOS	10 g sc-FOS or maltodextrin (control), daily for 8 days, followed by intermittent consumption up to 36 days	Randomized, double-blind crossover design, DI; 10 g sc-FOS split between two 5 g supplements consumed at breakfast and dinner; 12 day washout period; habitual Ca intake was 316–858 mg/day	Adolescent girls from the Netherlands (*n* = 14), age 12–14 years	Fractional Ca and Mg absorption; circulating bone biomarkers, vit-D metabolites and related hormones; measures taken at 8 and 36 days	Ca and Mg absorption were unaffected at 8 days; after 36 days, despite intermittent consumption, Mg absorption was improved; after 36 days no effect of sc-FOS was observed for Ca absorption, bone turnover markers or vitamin D metabolites	van den Heuvel E, et al. 2009 (Ref. 124)
SCF	Diets containing 0, 10, and 20 g/day of SCF, for 50 days	Placebo-controlled crossover design, SI; 50 day washout; all participants provided with multivitamin-mineral with 200 mg Ca and 400 IU vit-D	Women, 4 + years postmenopausal (*n* = 12), age 40–78 years	Bone Ca retention by Ca-41; bone biochemical markers	Both 10 and 20 g/day of SCF improved whole-body Ca retention in a dose-dependent manner; bone alkaline phosphatase was the only biochemical marker of bone turnover that changed during the intervention resulting in a significant increase with 20 g/day SCF	Jackman S, et al. 2016 (Ref. 44)
SCF	0 and 12 g/day of SCF, for 3 weeks	Placebo-controlled crossover design (metabolic balance studies), DI; 7 day washout; Ca intake of 600 mg/day	Adolescent boys and girls, mixed race/ethnicity (*n* = 24), age 12–15 years	Fractional Ca absorption; calcium retention; bone biochemical markers and hormones were measured in urine or serum; phylogenetic diversity of bacterial communities by 16S rRNA sequencing	SCF significantly improved fractional Ca absorption but did not affect Ca retention; improvements in fractional Ca absorption were correlated with increases in *Bacteroides*, *Butyricicoccus*, *Oscillibacter* and *Dialister* genera but decreases in *Actinomyces*, *Pseudomonas*, and Other Erysipelotrichaceae genera; changes in biochemical markers of bone turnover were not observed	Whisner C, et al. 2014 (Ref. 42)
SCF	0, 10, and 20 g/day of SCF, for 4 weeks	Placebo-controlled crossover design, DI; 3 week washout; Ca intake of 800 mg/day during test days	Adolescent girls, Caucasian (*n* = 28), age 11–15 years	Fractional Ca absorption; bone biomarkers and hormones; phylogenetic diversity of bacterial communities were determined using 16S rRNA sequencing; fecal pH and SCFA concentrations	10 or 20 g/day SCF increased Ca absorption but the effect was not dose-dependent; diversity of fecal microbial communities increased with increasing SCF dose; increases in Ca absorption with 20 g/day were significantly correlated with increases in *Clostridium* and unclassified Clostridiaceae but microbes correlating with Ca absorption at this does were not consistent with those observed with 10 g/day SCF; SCFA did not differ by treatment but fecal pH was significantly lower with 20 g/day SCF when compared to 0 and 10 g/day treatments	Whisner C, et al. 2016 (Ref. 43)

*1,25(OH)*_2_ *D* 1,25 dihydroxy vitamin D, *BMC* bone mineral content, *BMD* bone mineral density, *BMI* body mass index, *Ca* calcium, *CTx* C-telopeptide of type I collagen, *Cu* copper, *DI* double-isotope method, *DNA* deoxyribose nucleic acid, *DXA* dual-energy X-ray absorptiometry, *Fe* iron, *FOS* fructooligosaccharides, *GOS* galactooligosaccharides, *IF* isoflavones, *ITF* inulin-type fructans, *Mg* magnesium, *OVX* ovariectomized, *P* phosphorus, *PCR-DGGE* polymerase chain reaction-density gradient gel electrophoresis, *PDX* polydextrose, *rRNA* ribosomal ribonucleic acid, *RS* resistant starch, *rt-qPCR* real-time quantitative polymerase chain reaction, *sc-FOS* short-chain fructooligosaccharides, *SCFA(s)* short-chain fatty acid(s), *SI* single-isotope method, *vit-D* vitamin D, *Zn* zinc

### Clinical Trials—Prebiotic Effects on Calcium Absorption and Mineral Balance

#### Effects of Age

Human prebiotic interventions have shown positive effects on mineral absorption, but these effects have varied some by age. While short-chain inulin at doses of 0.75–1.25 g/day improved iron and magnesium retention in infants 6–12 months of age, calcium and copper absorption were not affected at these doses [119]. Similarly, delivery of a 1:1 ratio of GOS and polydextrose (4 g/L) to formula-fed infants did not increase calcium absorption beyond that of formula-fed infants without prebiotic enrichment [120]. Both formula treatments (with and without prebiotics) in this study increased total absorbed calcium beyond that observed in human breastmilk-fed infants; however, the absorption efficiency was significantly greater from human milk when compared to both formulas. The larger quantity of calcium absorbed was likely due to the much higher calcium content of the formulas and should not be the reason to avoid breastfeeding as breastmilk has numerous benefits for infants including the ideal ratio of proteins, fats, vitamins, and immunological factors.

During the pubertal growth spurt, when poor calcium intakes can negatively influence attainment of peak bone mass, fructans, GOS, and SCF have been found to increase calcium absorption by 6–12% relative to control treatments in interventions ranging in length from 3 months to one year [42, 43, 47, 51, 121, 122]. These effects appear to persist throughout life, as consumption of lactulose, inulin, and GOS among postmenopausal women has resulted in significant increases in calcium absorption [27, 37, 52]. The menopausal transition is complex and presents an area for further investigation as little is known about how prebiotics influence calcium absorption in the early and late stages of menopause. One study of SCF that specifically focused on women during stable estrogen deficiency suggested that prebiotics improve calcium retention [44], while a study of utilizing 10 g/day short-chain FOS found that calcium absorption was improved in late-phase postmenopausal women but not early-phase postmenopausal women [123].

#### Prebiotic Type, Dose, and Duration Effects

Prebiotics have been shown to influence calcium absorption across a wide range of doses ranging from 8 to 20 g/day [27, 37, 42, 43, 47, 50, 51, 121, 122] with only a few studies showing no effect [123–125]. Calcium intake may explain differential effects on calcium absorption. In one of the null studies, calcium intakes (1500 mg/day) exceeded the recommended intake of 1300 mg/day suggesting that at high intakes the prebiotic effect may be overpowered by increased luminal calcium concentrations [125]. Conversely, another study with null effects may have been due to lower calcium intakes, but the authors also utilized a study design which lacked consistent consumption of the prebiotic. The latter findings suggest the importance of regular consumption in order to receive benefits to mineral absorption and bone health [124].

The long-term effects of prebiotic supplementation on calcium absorption have not been well studied as the majority of studies have followed participants for acute periods ranging from 9 days to 4 weeks. A slightly longer study with 6-week consumption of 10 g/day of both short- and long-chain inulin-type fructans observed significant increases in calcium and magnesium absorption among postmenopausal women [52]. Currently, the longest prebiotic consumption trial measuring calcium absorption was a 12-month evaluation of an inulin-type fructan mixture (8 g/day) in adolescent boys and girls [47]. This study measured calcium absorption at both 8 weeks and 12 months, and while absorption was significantly increased at both time points, a slight decrease was observed at 12 months relative to the earlier time point. Further, this study observed that the Fok1 genotype may influence early response to prebiotics which further suggests a need for longer term studies when non-responder effects are observed in the short term. Whether other prebiotics or variation in doses elicits similar responses over time remains severely understudied.

To date, dose–response studies on calcium absorption have been limited in human models. Among postmenopausal women, lactulose consumption at 10 g/day was successful at increasing calcium absorption in contrast to a 5 g/day treatment which did not differ from that of the control treatment [37]. In another study of postmenopausal women, SCF elicited a significant dose–response effect such that the 10-g treatment resulted in a 5% increase and the 20-g treatment a 7% increase in skeletal calcium retention [44]. The same fiber, when provided to adolescent boys and girls at the same doses, did not elicit a dose–response effect on calcium absorption; instead, both 10- and 20-g treatments resulted in approximately a 13% increase in absorption [43].

### Clinical Trials—Prebiotic Effects at the Level of Bone

#### Effects of Age

Human prebiotic studies that evaluate bone mineral density are extremely limited because long periods of time are required before changes in skeletal mineral can be seen by dual-energy X-ray absorptiometry, the current gold standard measurement technique. In the 12-month intervention of adolescents mentioned above, consumption of inulin-type fructans resulted in significantly greater whole-body bone mineral content (+ 35 g) and density (+ 0.015 g/cm^2^) [47]. Interestingly, this study also observed that approximately one-third of the study cohort did not respond to the prebiotic fiber, with responders (≥ 3% increase in calcium absorption relative to baseline) experiencing greater skeletal calcium accretion over the 12-month study when compared to non-responders (< 3% change in calcium absorption) and controls. The rare, long-lived (half-life of 10^5^ years) radioisotope, ^41^Ca, has been used to label the bone of postmenopausal women and study the effects of prebiotics on bone turnover. Daily supplementation with 50 g/day SCF resulted in improvements in calcium balance in the bone, as well as a significant increase (+ 8%) in bone alkaline phosphatase on the larger 20 g/day prebiotic dose [44].

#### Prebiotic Type, Dose, and Duration Effects

Shorter studies have also contributed to our understanding of bone tissue effects in humans, primarily by evaluating prebiotic-induced bone turnover changes. Among adolescent girls and boys, significant changes in biochemical markers of bone turnover were not observed but this may have been due to the high variance in these markers during such an intense period of growth [42]. Conversely, urinary concentrations of the resorption marker, deoxypyridinoline, were significantly lower in postmenopausal women consuming fructans [126]. The largest (*n* = 300) and longest duration known to have measured bone turnover markers, as well as bone mineral density, in postmenopausal women was 24 months [127]. This study found that 3.6 g/day short-chain FOS + 800 mg/day calcium consumption (CaFOS) decreased serum concentrations of C-telopeptides of type I collagen at 12 months and osteocalcin at 24 months to a greater degree relative to maltodextrin (9.8 g/day) control. While this suggested that CaFOS reduced bone turnover better than other treatments, significant preservation of total body bone mineral density was only seen in the CaFOS group compared to calcium (800 mg/day) alone. Further, this study suggested a greater benefit to women with osteopenia which highlights the importance of metabolic condition when evaluating the influence of prebiotics on bone health.

### Clinical Trials—Influence of Gut Microbes on Prebiotic-Induced Bone Effects

Quantitative PCR has been used more regularly to assess changes in specific microbial groups in relation to prebiotic treatment, but this method does not take into account changes in the overall microbial community. High-throughput sequencing of the conserved 16S ribosomal RNA gene now allows for assessment of changes in microbial diversity, as well as changes down to the genus level across the entire microbial community. This sequencing method has begun to allow researchers to answer microbially related mechanistic questions in calcium metabolism studies. To date, only a few studies have implemented these methods in prebiotic–mineral absorption studies.

Changes in the gut microbial composition have been associated with improvements in calcium absorption in adolescent girls and boys consuming 12 g/day of SCF [42]. Specific genera that increased with SCF consumption were *Bacteroides*, *Butyricicoccus*, *Oscillibacte*r, and *Dialister*. In a dose–response study using the same fiber at 10 and 20 g/day doses, improvements in calcium absorption among Caucasian adolescent girls correlated significantly with a number of microbial genera known to ferment prebiotic dietary fibers [43]. Additionally, diversity of the fecal microbial community was also increased in this study. Specific changes in the intestinal microbial community were increased proportions of *Parabacteroides* and *Clostridium*. Overall, these preliminary studies in humans suggest the importance of microbial community shifts in contributing to improved mineral absorption, but further research is needed to elucidate causal pathways and confirm that findings are relevant to other populations and prebiotic fibers. To date, fecal microbial analysis has not been extensively studied in postmenopausal women in relation to measures of calcium or bone metabolism.

## Prebiotics as a Dietary Strategy for Osteoporosis Prevention and Treatment

Two primary strategies exist for promoting bone health across the lifespan. Earlier in life, the best strategy is to engage in behaviors that promote the attainment of peak bone mass. Once this peak has been achieved, the strategy must shift toward one of maintaining and reducing the loss of bone. Despite substantial evidence that calcium consumption is critical for optimal bone health and reducing fracture risk later, Americans do not consume adequate amounts of calcium [128]. Prebiotics are an alternative and effective method for increasing calcium absorption and bone mineral density among individuals with inadequate calcium intakes. They also appear to reduce the rate of bone loss and reduce fracture risk in later life making them an effective public health approach for older individuals as well. Overall, the benefits of prebiotics are modest and should not be viewed as a singular approach for bone health, especially among individuals with gross deficiencies of calcium intake.

Fiber has long been known to influence metabolic diseases in a beneficial manner, but the current literature only reports beneficial impacts of these fibers when extracted or delivered in more concentrated doses. While extracted prebiotics have proven beneficial for calcium absorption when delivered to humans in a variety of foods ranging from baked goods, drinks, smoothies, and juices [42, 44, 47, 121], no studies, to our knowledge, have evaluated the effects of fiber-rich diets as a means to increase prebiotic intake and subsequent bone health outcomes. As mentioned earlier in this review, a variety of foods including grains, vegetables, and some fruits naturally contain prebiotic fibers. How the matrices of these foods influence prebiotic availability and digestibility by intestinal microbes remains largely unanswered. Currently, the largest contributor of inulin and oligofructose in the American diet is wheat products [129]. Wheat also contains compounds, especially in the bran fraction, which have been shown to decrease calcium bioavailability [130]. More recent research with regard to intestinal microbiota effects suggests that arabinoxylan oligosaccharide-rich wheat bran extracts and whole-grain wheat may increase the composition of bifidobacteria and lactobacillus [131, 132], which have been linked to improvements in bone health. If prebiotics elicit their benefit to bone through actions of the gut microbiota, dietary approaches to incorporate these functional ingredients or foods naturally containing prebiotics should be a focus of bone health recommendations.

## Conclusions and Areas for Further Research

The study of prebiotic effects on calcium metabolism and bone health is a rapidly growing area of investigation. The majority of current evidence suggests their beneficial impact during periods of rapid adolescent growth and also life stages characterized with greater bone mineral loss and fracture risk. A primary concept for prebiotic effectiveness is their ability to resist digestion by human enzymes, but little is known about how the source and structure of these compounds influence the overall community and specific microbial members to elicit benefits to bone.

To date, research has focused primarily on bifidobacteria counts, with disaccharide (e.g., lactulose and difructose anhydrides), oligosaccharide (e.g., short-chain FOS, GOS), and polysaccharide (e.g., long-chain FOS and inulin) prebiotics altering the microbial content of the intestine in different ways. Further, it appears that prebiotic type differentially influences the absorption of different minerals and bone mineral and strength parameters. Therefore, further study to clearly elucidate the unique skeletal benefits of prebiotic fibers (sugar monomer composition, degree of polymerization, branching, bonds, etc.) is needed. Further, comparison of whole-food dietary approaches with currently used supplementation approaches would expand our ability to identify specific foods and fiber types that are most beneficial for bone health. Future research should also strive to understand the long-term effects of prebiotics and how different doses influence outcomes, as this will be important for future dietary and clinical recommendations.

The most exciting of future research efforts will be those that continue to characterize gut microbial communities but also evaluate microbial metabolism beyond SCFAs. Specifically, the study of signaling molecules on metagenomic, metabolomic, and epigenetic pathways will greatly contribute to current mechanistic understandings. Further exploration in these areas will ultimately allow clinicians to manipulate the gut microbiome for optimal bone health and possibly avoid the use of pharmaceuticals with adverse side effects for the treatment of osteoporosis.

## References

[1] Ly NP, Litonjua A, Gold DR, Celedón JC (2011). Gut microbiota, probiotics, and vitamin D: interrelated exposures influencing allergy, asthma, and obesity?. J Allergy Clin Immunol.

[2] Sanz Y (2011). Gut microbiota and probiotics in maternal and infant health. Am J Clin Nutr.

[3] Flint HJ (2012). The impact of nutrition on the human microbiome. Nutr Rev.

[4] Villa CR, Ward WE, Comelli EM (2017). Gut microbiota-bone axis. Crit Rev Food Sci Nutr.

[5] McCabe L, Britton RA, Parameswaran N (2015). Prebiotic and probiotic regulation of bone health: role of the intestine and its microbiome. Curr Osteoporos Rep.

[6] Weaver CM (2015). Diet, gut microbiome, and bone health. Curr Osteoporos Rep.

[7] Gibson GR, Scott KP, Rastall RA (2010). Dietary prebiotics: current status and new definition. Food Sci Technol Bull Funct Foods.

[8] Bindels LB, Delzenne NM, Cani PD, Walter J (2015). Towards a more comprehensive concept for prebiotics. Nat Rev Gastroenterol Hepatol.

[9] Slavin J (2013). Fiber and prebiotics: mechanisms and health benefits. Nutrients.

[10] Gibson GR, Roberfroid MB (1995). Dietary modulation of the human colonic microbiota: introducing the concept of prebiotics. J Nutr.

[11] Leach JD, Sobolik KD (2010). High dietary intake of prebiotic inulin-type fructans in the prehistoric Chihuahuan Desert. Br J Nutr.

[12] Van Loo J, Coussement P, De Leenheer L (1995). On the presence of Inulin and Oligofructose as natural ingredients in the western diet. Crit Rev Food Sci Nutr.

[13] Scholz-Ahrens KE, Weaver CM, Daly RM, Bischoff-Ferrari HA (2016). Prebiotics, probiotics, synbiotics and foods with regard to bone metabolism. Nutritional influences on bone health.

[14] Roberfroid M, Gibson GR, Hoyles L (2010). Prebiotic effects: metabolic and health benefits. Br J Nutr.

[15] Roberfroid MB (2007). Inulin-type fructans: functional food ingredients. J Nutr.

[16] Sabater-Molina M, Larqué E, Torrella F, Zamora S (2009). Dietary fructooligosaccharides and potential benefits on health. J Physiol Biochem.

[17] Kelly G (2008). Inulin-type prebiotics—a review: part 1. Altern Med Rev.

[18] Carabin IG, Flamm WG (1999). Evaluation of safety of inulin and oligofructose as dietary fiber. Regul Toxicol Pharmacol.

[19] Bode L (2006). Recent advances on structure, metabolism, and function of human milk oligosaccharides. J Nutr.

[20] Fanaro S, Boehm G, Garssen J (2007). Galacto-oligosaccharides and long-chain fructo-oligosaccharides as prebiotics in infant formulas: a review. Acta Paediatr.

[21] Torres DPM, Gonçalves MDPF, Teixeira JA, Rodrigues LR (2010). Galacto-oligosaccharides: production, properties, applications, and significance as prebiotics. Compr Rev Food Sci Food Saf.

[22] Hernandez-Hernandez O, Marin-Manzano MC, Rubio LA (2012). Monomer and linkage type of galacto-oligosaccharides affect their resistance to ileal digestion and prebiotic properties in rats. J Nutr.

[23] van Leeuwen SS, Kuipers BJH, Dijkhuizen L, Kamerling JP (2016). Comparative structural characterization of 7 commercial galacto-oligosaccharide (GOS) products. Carbohydr Res.

[24] Chonan O, Matsumoto K, Watanuki M (1995). Effect of galactooligosaccharides on calcium absorption and preventing bone loss in ovariectomized rats. Biosci Biotechnol Biochem.

[25] Chonan O, Watanuki M (1996). The effect of 6′-galactooligosaccharides on bone mineralization of rats adapted to different levels of dietary calcium. Int J Vitam Nutr Res.

[26] Weaver CM, Martin BR, Nakatsu CH (2011). Galactooligosaccharides improve mineral absorption and bone properties in growing rats through gut fermentation. J Agric Food Chem.

[27] van den Heuvel E, Schoterman M, Muijs T (2000). Trans-galactooligosaccharides stimulate calcium absorption in postmenopausal women. J Nutr.

[28] Schaafsma G, Visser WJ, Dekker PR, Van Schaik M (1987). Effect of dietary calcium supplementation with lactose on bone in vitamin D-deficient rats. Bone.

[29] Misselwitz B, Pohl D, Frühauf H (2013). Lactose malabsorption and intolerance: pathogenesis, diagnosis and treatment. United Eur Gastroenterol J.

[30] Griessen M, Cochet B, Infante F (1989). Calcium absorption from milk in lactase-deficient subjects. Am J Clin Nutr.

[31] Cochet B, Jung A, Griessen M (1960). Effects of lactose on intestinal calcium absorption in normal and lactase-deficient subjects. Gastroenterology.

[32] Tremaine WJ, Newcomer AD, Riggs BL, McGill DB (1986). Calcium absorption from milk in lactase-deficient and lactase-sufficient adults. Dig Dis Sci.

[33] Szilagyi A (2002). Review article: lactose–a potential prebiotic. Aliment Pharmacol Ther.

[34] Pranami D, Sharma R, Pathak H (2017). Lactulose: a prebiotic, laxative and detoxifying agent. Drugs Ther Perspect.

[35] Aider M, de Halleux D (2007). Isomerization of lactose and lactulose production: review. Trends Food Sci Technol.

[36] Brommage R, Binacua C, Antille S, Carrié AL (1993). Intestinal calcium absorption in rats is stimulated by dietary lactulose and other resistant sugars. J Nutr.

[37] van den Heuvel EGHM, Muijs T, van Dokkum W, Schaafsma G (1999). Lactulose stimulates calcium absorption in postmenopausal women. J Bone Miner Res.

[38] Tate & Lyle (2014) Soluble corn fiber: health benefits and product applications. http://www.foodnutritionknowledge.info/. Accessed 30 Aug 2016

[39] Costabile A, Deaville ER, Morales AM (2016). Prebiotic potential of a maize-based soluble fibre and impact of dose on the human gut microbiota. PLoS ONE.

[40] Housez B, Cazaubiel M, Vergara C (2012). Evaluation of digestive tolerance of a soluble corn fibre. J Hum Nutr Diet.

[41] Weaver CM, Martin BR, Story JA (2010). Novel fibers increase bone calcium content and strength beyond efficiency of large intestine fermentation. J Agric Food Chem.

[42] Whisner CM, Martin BR, Nakatsu CH (2014). Soluble maize fibre affects short-term calcium absorption in adolescent boys and girls: a randomised controlled trial using dual stable isotopic tracers. Br J Nutr.

[43] Whisner CM, Martin BR, Nakatsu CH (2016). Soluble corn fiber increases calcium absorption associated with shifts in the gut microbiome: a randomized dose-response trial in free-living pubertal females. J Nutr.

[44] Jakeman SA, Henry CN, Martin BR (2016). Soluble corn fiber increases bone calcium retention in postmenopausal women in a dose-dependent manner: a randomized crossover trial. Am J Clin Nutr.

[45] Pandey KR, Naik SR, Vakil BV (2015). Probiotics, prebiotics and synbiotics—a review. J Food Sci Technol.

[46] Coxam V (2005). Inulin-type fructans and bone health: state of the art and perspectives in the management of osteoporosis. Br J Nutr.

[47] Abrams SA, Griffin IJ, Hawthorne KM (2005). A combination of prebiotic short- and long-chain inulin-type fructans enhances calcium absorption and bone mineralization in young adolescents. Am J Clin Nutr.

[48] Abrams SA, Griffin IJ, Hawthorne KM (2007). Young adolescents who respond to an inulin-type fructan substantially increase total absorbed calcium and daily calcium accretion to the skeleton. J Nutr.

[49] Abrams SA, Hawthorne KM, Aliu O (2007). An inulin-type fructan enhances calcium absorption primarily via an effect on colonic absorption in humans. J Nutr.

[50] Griffin IJ, Davila PM, Abrams SA (2002). Non-digestible oligosaccharides and calcium absorption in girls with adequate calcium intakes. Br J Nutr.

[51] Griffin IJ, Hicks PM, Heaney RP (2003). Enriched chicory inulin increases calcium absorption mainly in girls with lower calcium absorption. Nutr Res.

[52] Holloway L, Moynihan S, Abrams SA (2007). Effects of oligofructose-enriched inulin on intestinal absorption of calcium and magnesium and bone turnover markers in postmenopausal women. Br J Nutr.

[53] Bryk G, Coronel MZ, Pellegrini G (2015). Effect of a combination GOS/FOS^®^ prebiotic mixture and interaction with calcium intake on mineral absorption and bone parameters in growing rats. Eur J Nutr.

[54] Devareddy L, Khalil DA, Korlagunta K (2006). The effects of fructo-oligosaccharides in combination with soy protein on bone in osteopenic ovariectomized rats. Menopause.

[55] Johnson CD, Lucas EA, Hooshmand S (2011). Addition of fructooligosaccharides and dried plum to soy-based diets reverses bone loss in the ovariectomized rat. Evid Based Complement Alternat Med.

[56] Rodrigues FC, Castro ASB, Rodrigues VC (2012). Yacon flour and *Bifidobacterium longum* modulate bone health in rats. J Med Food.

[57] Pérez-Conesa D, López G, Abellán P, Ros G (2006). Bioavailability of calcium, magnesium and phosphorus in rats fed probiotic, prebiotic and synbiotic powder follow-up infant formulas and their effect on physiological and nutritional parameters. J Sci Food Agric.

[58] Pérez-Conesa D, López G, Ros G (2007). Effects of probiotic, prebiotic and synbiotic follow-up infant formulas on large intestine morphology and bone mineralisation in rats. J Sci Food Agric.

[59] Adolphi B, Scholz-Ahrens KE, de Vrese M (2009). Short-term effect of bedtime consumption of fermented milk supplemented with calcium, inulin-type fructans and caseinphosphopeptides on bone metabolism in healthy, postmenopausal women. Eur J Nutr.

[60] Tu M-Y, Chen H-L, Tung Y-T (2015). Short-term effects of kefir-fermented milk consumption on bone mineral density and bone metabolism in a randomized clinical trial of osteoporotic patients. PLoS ONE.

[61] Kruger MC, Chan YM, Kuhn-Sherlock B (2016). Differential effects of calcium- and vitamin D-fortified milk with FOS-inulin compared to regular milk, on bone biomarkers in Chinese pre- and postmenopausal women. Eur J Nutr.

[62] Lam K-L, Chi-Keung Cheung P (2013). Non-digestible long chain beta-glucans as novel prebiotics. Bioact Carbohydr Diet Fibre.

[63] Reilly P, Sweeney T, O’Shea C (2010). The effect of cereal-derived beta-glucans and exogenous enzyme supplementation on intestinal microflora, nutrient digestibility, mineral metabolism and volatile fatty acid concentrations in finisher pigs. Anim Feed Sci Technol.

[64] Broekaert WF, Courtin CM, Verbeke K (2011). Prebiotic and other health-related effects of cereal-derived arabinoxylans, arabinoxylan-oligosaccharides, and xylooligosaccharides. Crit Rev Food Sci Nutr.

[65] Cloetens L, Broekaert WF, Delaedt Y (2010). Tolerance of arabinoxylan-oligosaccharides and their prebiotic activity in healthy subjects: a randomised, placebo-controlled cross-over study. Br J Nutr.

[66] Lugani Y, Sooch S (2017). Xylitol, an emerging prebiotic: a review. Int J Appl Pharm Biol Res.

[67] Qing Q, Li H, Kumar R, Wyman CE, Wyman CE (2013). Xylooligosaccharides production, quantification, and characterization in context of lignocellulosic biomass pretreatment. Aqueous pretreatment of plant biomass for biological and chemical conversion to fuels and chemicals.

[68] Campbell JM, Fahey GC, Wolf BW (1997). Selected indigestible oligosaccharides affect large bowel mass, cecal and fecal short-chain fatty acids, pH and microflora in rats. J Nutr.

[69] Wang S, Zhang P, Kong X (2017). Delicate changes of bioapatite mineral in pig femur with addition of dietary xylooligosaccharide: evidences from Raman spectroscopy and ICP. Anim Sci J.

[70] Qin J, Li R, Raes J (2010). A human gut microbial gene catalogue established by metagenomic sequencing. Nature.

[71] Zheng X, Xie G, Zhao A (2011). The footprints of gut microbial-mammalian co-metabolism. J Proteome Res.

[72] Scholz-Ahrens KE, Ade P, Marten B (2007). Prebiotics, probiotics, and synbiotics affect mineral absorption, bone mineral content, and bone structure. J Nutr.

[73] Scholz-Ahrens KE, Schrezenmeir J (2002). Inulin, oligofructose and mineral metabolism—experimental data and mechanism. Br J Nutr.

[74] Sjögren K, Engdahl C, Henning P (2012). The gut microbiota regulates bone mass in mice. J Bone Miner Res.

[75] Scholz-Ahrens KE, Adolphi B, Rochat F (2016). Effects of probiotics, prebiotics, and synbiotics on mineral metabolism in ovariectomized rats—impact of bacterial mass, intestinal absorptive area and reduction of bone turn-over. NFS J.

[76] Tousen Y, Abe F, Ishida T (2011). Resistant starch promotes equol production and inhibits tibial bone loss in ovariectomized mice treated with daidzein. Metabolism.

[77] Tousen Y, Matsumoto Y, Matsumoto C (2016). The combined effects of soya isoflavones and resistant starch on equol production and trabecular bone loss in ovariectomised mice. Br J Nutr.

[78] Yang L-C, Wu J-B, Lu T-J, Lin W-C (2013). The prebiotic effect of *Anoectochilus formosanus* and its consequences on bone health. Br J Nutr.

[79] Raschka L, Daniel H (2005). Mechanisms underlying the effects of inulin-type fructans on calcium absorption in the large intestine of rats. Bone.

[80] García-Vieyra MI, Del Real A, López MG (2014). Agave fructans: their effect on mineral absorption and bone mineral content. J Med Food.

[81] Mineo H, Hara H, Tomita F (2001). Short-chain fatty acids enhance diffusional ca transport in the epithelium of the rat cecum and colon. Life Sci.

[82] Donohoe DR, Garge N, Zhang X (2011). The microbiome and butyrate regulate energy metabolism and autophagy in the mammalian colon. Cell Metab.

[83] Scheppach W, Bartram P, Richter A (1992). Effect of short-chain fatty acids on the human colonic mucosa in vitro. JPEN J Parenter Enteral Nutr.

[84] Ohta A, Motohashi Y, Sakai K (1998). Dietary fructooligosaccharides increase calcium absorption and levels of mucosal calbindin-D9k in the large intestine of gastrectomized rats. Scand J Gastroenterol.

[85] Henagan T, Navard A, Ye J (2014). Sodium butyrate remodels whole genome nucleosome maps and attenuates high fat diet-induced mitochondrial dysfunction in skeletal muscle from C57BL6/J mice (1072.1). FASEB J.

[86] Macpherson AJ, Harris NL (2004). Interactions between commensal intestinal bacteria and the immune system. Nat Rev Immunol.

[87] Irwin R, Lee T, Young VB (2013). Colitis-induced bone loss is gender dependent and associated with increased inflammation. Inflamm Bowel Dis.

[88] Yadav VK, Ryu J-H, Suda N (2008). Lrp5 controls bone formation by inhibiting serotonin synthesis in the duodenum. Cell.

[89] Yun H-M, Park K-R, Hong JT (2016). Peripheral serotonin-mediated system suppresses bone development and regeneration via serotonin 6 G-protein-coupled receptor. Sci Rep.

[90] Yadav VK, Balaji S, Suresh PS (2010). Pharmacological inhibition of gut-derived serotonin synthesis is a potential bone anabolic treatment for osteoporosis. Nat Med.

[91] Coudray C, Feillet-Coudray C, Tressol JC (2005). Stimulatory effect of inulin on intestinal absorption of calcium and magnesium in rats is modulated by dietary calcium intakes short- and long-term balance studies. Eur J Nutr.

[92] Krupa-Kozak U, Markiewicz L, Lamparski G, Juśkiewicz J (2017). Administration of inulin-supplemented gluten-free diet modified calcium absorption and caecal microbiota in rats in a calcium-dependent manner. Nutrients.

[93] Suzuki T, Hara H (2004). Various nondigestible saccharides open a paracellular calcium transport pathway with the induction of intracellular calcium signaling in human intestinal Caco-2 cells. J Nutr.

[94] Krupa-Kozak U, Swiątecka D, Bączek N, Brzóska MM (2016). Inulin and fructooligosaccharide affect in vitro calcium uptake and absorption from calcium-enriched gluten-free bread. Food Funct.

[95] Gilman J, Cashman KD (2006). The effect of probiotic bacteria on transepithelial calcium transport and calcium uptake in human intestinal-like Caco-2 cells. Curr Issues Intest Microbiol.

[96] Roberfroid MB, Cumps J, Devogelaer JP (2002). Dietary chicory inulin increases whole-body bone mineral density in growing male rats. J Nutr.

[97] Legette LL, Lee W, Martin BR (2012). Prebiotics enhance magnesium absorption and inulin-based fibers exert chronic effects on calcium utilization in a postmenopausal rodent model. J Food Sci.

[98] Coudray C, Tressol JC, Gueux E, Rayssiguier Y (2003). Effects of inulin-type fructans of different chain length and type of branching on intestinal absorption and balance of calcium and magnesium in rats. Eur J Nutr.

[99] Levrat MA, Rémésy C, Demigné C (1991). High propionic acid fermentations and mineral accumulation in the cecum of rats adapted to different levels of inulin. J Nutr.

[100] Coudray C, Rambeau M, Feillet-Coudray C (2005). Dietary inulin intake and age can significantly affect intestinal absorption of calcium and magnesium in rats: a stable isotope approach. Nutr J.

[101] Ohta A, Motohashi Y, Ohtsuki M (1998). Dietary fructooligosaccharides change the concentration of calbindin-D9k differently in the mucosa of the small and large intestine of rats. J Nutr.

[102] Takasaki M, Inaba H, Ohta A (2000). Dietary short-chain fructooligosaccharides increase calbindin-D9k levels only in the large intestine in rats independent of dietary calcium deficiency or serum 1,25 dihydroxy vitamin D levels. Int J Vitam Nutr Res.

[103] Ohta A, Ohtsuki M, Baba S (1998). Comparison of the nutritional effects of fructo-oligosaccharides of different sugar chain length in rats. Nutr Res.

[104] Coudray C, Feillet-Coudray C, Gueux E (2006). Dietary inulin intake and age can affect intestinal absorption of zinc and copper in rats. J Nutr.

[105] Zafar TA, Weaver CM, Zhao Y (2004). Nondigestible oligosaccharides increase calcium absorption and suppress bone resorption in ovariectomized rats. J Nutr.

[106] Mitamura R, Hara H (2006). Ingestion of difructose anhydride III partially restores calcium absorption impaired by vitamin D and estrogen deficiency in rats. Eur J Nutr.

[107] Ohta A, Ohtuki M, Takizawa T (1994). Effects of fructooligosaccharides on the absorption of magnesium and calcium by cecectomized rats. Int J Vitam Nutr Res.

[108] Scholz-Ahrens KE, Açil Y, Schrezenmeir J (2002). Effect of oligofructose or dietary calcium on repeated calcium and phosphorus balances, bone mineralization and trabecular structure in ovariectomized rats. Br J Nutr.

[109] Morohashi T, Sano T, Ohta A, Yamada S (1998). True calcium absorption in the intestine is enhanced by fructooligosaccharide feeding in rats. J Nutr.

[110] Ohta A, Ohtsuki M, Baba S (1995). Calcium and magnesium absorption from the colon and rectum are increased in rats fed fructooligosaccharides. J Nutr.

[111] Lobo AR, Filho JM, Alvares EP (2009). Effects of dietary lipid composition and inulin-type fructans on mineral bioavailability in growing rats. Nutrition.

[112] Takahara S, Morohashi T, Sano T (2000). Fructooligosaccharide consumption enhances femoral bone volume and mineral concentrations in rats. J Nutr.

[113] Lobo AR, Colli C, Filisetti TMCC (2006). Fructooligosaccharides improve bone mass and biomechanical properties in rats. Nutr Res.

[114] Demigné C, Jacobs H, Moundras C (2008). Comparison of native or reformulated chicory fructans, or non-purified chicory, on rat cecal fermentation and mineral metabolism. Eur J Nutr.

[115] Mathey J, Puel C, Kati-Coulibaly S (2004). Fructooligosaccharides maximize bone-sparing effects of soy isoflavone-enriched diet in the ovariectomized rat. Calcif Tissue Int.

[116] Zafar TA, Weaver CM, Jones K (2004). Inulin effects on bioavailability of soy isoflavones and their calcium absorption enhancing ability. J Agric Food Chem.

[117] Ohta A, Uehara M, Sakai K (2002). A combination of dietary fructooligosaccharides and isoflavone conjugates increases femoral bone mineral density and equol production in ovariectomized mice. J Nutr.

[118] Legette LL, Lee W-H, Martin BR (2011). Genistein, a phytoestrogen, improves total cholesterol, and Synergy, a prebiotic, improves calcium utilization, but there were no synergistic effects. Menopause.

[119] Yap KW, Mohamed S, Yazid AM (2005). Dose-response effects of inulin on the faecal short-chain fatty acids content and mineral absorption of formula-fed infants. Nutr Food Sci.

[120] Hicks PD, Hawthorne KM, Berseth CL (2012). Total calcium absorption is similar from infant formulas with and without prebiotics and exceeds that in human milk-fed infants. BMC Pediatr.

[121] Whisner CM, Martin BR, Schoterman MHC (2013). Galacto-oligosaccharides increase calcium absorption and gut bifidobacteria in young girls: a double-blind cross-over trial. Br J Nutr.

[122] van den Heuvel EGHM, Muys T, van Dokkum W, Schaafsma G (1999). Oligofructose stimulates calcium absorption in adolescents. Am J Clin Nutr.

[123] Tahiri M, Tressol JC, Arnaud J (2003). Effect of short-chain fructooligosaccharides on intestinal calcium absorption and calcium status in postmenopausal women: a stable-isotope study. Am J Clin Nutr.

[124] van den Heuvel EGHM, Muijs T, Brouns F, Hendriks HFJ (2009). Short-chain fructo-oligosaccharides improve magnesium absorption in adolescent girls with a low calcium intake. Nutr Res.

[125] Martin BR, Braun MM, Wigertz K (2010). Fructo-oligosaccharides and calcium absorption and retention in adolescent girls. J Am Coll Nutr.

[126] Kim Y-Y, Jang K-H, Lee E-Y (2004). The effect of chicory fructan fiber on calcium absorption and bone metabolism in Korean postmenopausal women. Nutr Sci.

[127] Slevin MM, Allsopp PJ, Magee PJ (2014). Supplementation with calcium and short-chain fructo-oligosaccharides affects markers of bone turnover but not bone mineral density in postmenopausal women. J Nutr.

[128] Scientific Report of the 2015 Dietary Guidelines Advisory Committee. http://www.health.gov/dietaryguidelines/2015-scientific-report/. Accessed 23 Jul 2015

[129] Moshfegh AJ, Friday JE, Goldman JP, Ahuja JK (1999). Presence of inulin and oligofructose in the diets of Americans. J Nutr.

[130] Weaver CM, Heaney RP, Teegarden D, Hinders SM (1996). Wheat bran abolishes the inverse relationship between calcium load size and absorption fraction in women. J Nutr.

[131] François IEJA, Lescroart O, Veraverbeke WS (2014). Effects of wheat bran extract containing arabinoxylan oligosaccharides on gastrointestinal parameters in healthy preadolescent children. J Pediatr Gastroenterol Nutr.

[132] Costabile A, Klinder A, Fava F (2008). Whole-grain wheat breakfast cereal has a prebiotic effect on the human gut microbiota: a double-blind, placebo-controlled, crossover study. Br J Nutr.

